# Crystallization-Induced Self-Assembly of Poly(ethylene glycol) Side Chains in Dithiol–yne-Based Comb Polymers: Side Chain Spacing and Molecular Weight Effects

**DOI:** 10.1021/acs.macromol.4c00527

**Published:** 2024-05-15

**Authors:** Eider Matxinandiarena, Mario Iván Peñas, Brennan J. Curole, Monika Król, Lucas Polo Fonseca, Janne Ruokolainen, Scott M. Grayson, Leire Sangroniz, Alejandro J. Müller

**Affiliations:** † POLYMAT and Department of Polymers and Advanced Materials: Physics, Chemistry and Technology, Faculty of Chemistry, University of the Basque Country UPV/EHU, Paseo Manuel de Lardizábal, 3, 20018 Donostia-San Sebastián, Spain; ‡ Department of Chemistry, Tulane University, 6400 Freret Street, 2015 Percival Stern Hall, New Orleans, Louisiana 70118, United States; § Department of Applied Physics, School of Science, Aalto University, FIN-00076 Espoo, Finland; ∥ IKERBASQUE, Basque Foundation for Science, Plaza Euskadi 5, 48009 Bilbao, Spain

## Abstract

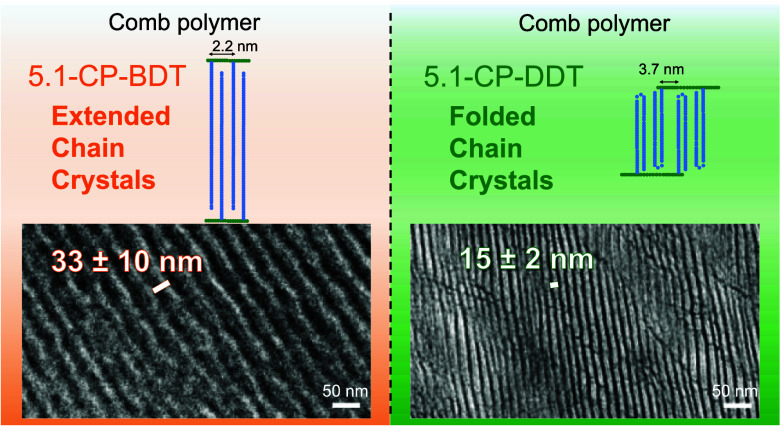

The chain architecture and topology of macromolecules impact their physical properties and final performance, including their crystallization process. In this work, comb polymers constituted by poly(ethylene glycol), PEG, side chains, and a dithiol–yne-based ring polymer backbone have been studied, focusing on the micro- and nanostructures of the system, thermal behavior, and crystallization kinetics. The designed comb system allows us to investigate the role of a ring backbone, the impact of varying the distance between two neighboring side chains, and the effect of the molecular weight of the side chain. The results reflect that the governing factor in the crystalline properties is the molar mass of the side chains and that the tethering of PEG chains to the ring backbone brings important constraints to the crystallization process, reducing the crystallinity degree and slowing down the crystallization kinetics in comparison to analogue PEG homopolymers. We demonstrate that the effect of spatial hindrance in the comb-like PEG polymers drives the morphology toward highly ordered, self-assembled, semicrystalline superstructures with either extended interdigitated chain crystals or novel (for comb polymers) interdigitated folded chain lamellar crystals. These structures depend on PEG molecular weight, the distance between neighboring tethered PEG chains, and the crystallization conditions (nonisothermal versus isothermal). This work sheds light on the role of chain architecture and topology in the structure of comb-like semicrystalline polymers.

## 1 Introduction

Poly(ethylene glycol) (PEG) is a thermoplastic polymer with a relatively simple chemical structure. It is semicrystalline, water-soluble, and biocompatible.^1,2^ It has good lubrication properties, appropriate water retention, and low toxicity. Depending on the molar mass, the melting temperature of PEG varies from 0 to 65 °C.^3^ Thus, low-molar-mass samples are liquid at room temperature, whereas high-molar-mass samples are solid. As a result, PEG can display a wide range of physical properties, which enables this material to have many applications, including cosmetics, pharmaceutics, drug delivery, polymer electrolytes, or mining.^1,2^

An exciting strategy to modify and control the physical properties of polymers is the design and precise control of polymer architectures with new topologies such as cyclic, star, H-shaped, comb, or brush polymers. These new topologies bring unique properties with upgraded performance, such as higher thermal stability, reduced viscosity, or improved solubility.^4,5^ Comb-like polymers have a peculiar chain topology formed by a linear backbone and pending side chains. In this case, one end of the side chain is tethered to the polymer backbone. When the grafting density of side chains is low, the materials are called comb polymers, whereas the materials with a high density of side chains are known as bottlebrushes. Comb polymers can be defined with three independent parameters: the molecular weight of the main chain or backbone, the molecular weight of the side chains, and the number of side chains per backbone. Another relevant parameter is the branch point spacing, i.e., the distance between two neighboring side chains.^6,7^ These materials have applications in antifouling, drug delivery systems, antibacterials, and batteries.^4,5^

To control and tailor the final performance of these branched materials, it is necessary to understand the structure of comb polymers. It has been shown that the branch point distribution along the chain affects, among others, the rheological properties in low graft density comb polymers, broadening the relaxation time. Thus, designing synthetic methods to control the space between side chains is desirable to have well-defined properties, but it remains challenging.^6,7^ Recently, a new strategy to synthesize comb polymers with precise control of the length of the side chains and the space between side chains has been reported by Curole et al.,^8^ employing dithiol–yne “click chemistry” to obtain polythioether macrocycles based on several alkanedithiol backbone spacers. With this technique, novel macrocyclic comb polymers with PEG side chains have been synthesized.

**Figure 1:**
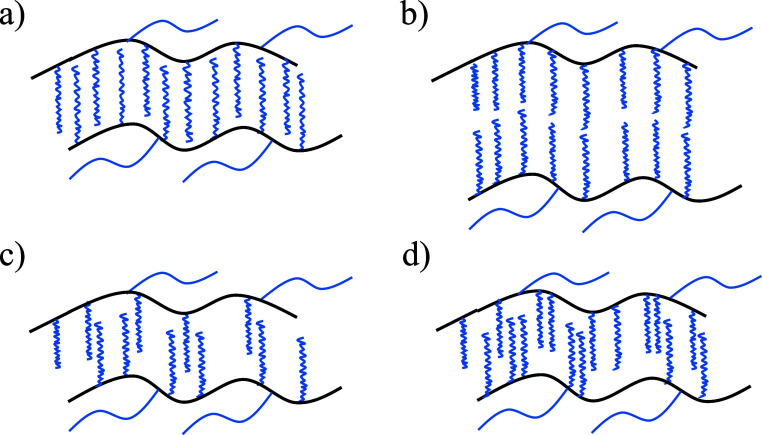
Scheme representing the interdigitated packing mode of the chains in comb polymers based on ref (2). (a) Entirely interdigitated, (b) end-to-end bimolecular layer, (c,d) partially interdigitated cases.

As has been mentioned, the topology of the chain can significantly impact the physical properties such as rheology, mechanical performance, or crystallization properties. Considering the role of crystallinity on final properties such as permeability, degradation, or mechanical performance, it is of great relevance to understand the implications of chain architecture and topology on the thermal behavior of the materials and to establish structure–property relationships. It is well-known that the side chains can crystallize in comb polymers, whereas the polymer backbone is usually rejected to the amorphous phase. The crystallization of side chains will depend on backbone flexibility, grafting density, and side chain length.^9^ Several studies have been carried out with comb polymers constituted by flexible backbones, determining the minimum length of the side chain to form the crystals.^9−11^ On the other hand, the study of their semicrystalline structure has shown that several systems pack in an interdigitated manner, see Figure 1. The detailed study of PEI-octadecanoic acid and other comb polymers revealed that the variation in the composition could change the packing, making possible the entirely interdigitated packing of chains, partially interdigitated, or end-to-end bimolecular layers.^1,2^

In recent years, a few comb and brush polymers with PEG side chains have been investigated, such as comb polymers containing PEG side chains and a crystallizable hydrocarbon segment in the backbone, which forms 2 crystalline phases, one containing PEG chains and the other the hydrocarbon segments. These polymers contain several ester-based segments in the repeating unit of the backbone. Sarkar et al. proved that by increasing the number of methylene groups in one of the ester segments, the interlamellar spacing of the system increases. Chanda et al. have proven with a similar system that it is possible to control the dimensions of the interlamellar spacing of PEG side chains lamellae. The mentioned works have focused on the morphology and structure of the system.^12,13^

Other works have centered on the thermal properties,^14−16^ such as brush polymer with a poly(norbornene) backbone and PEG side chains of several lengths (3 and 6 kg/mol).^14^ The brush polymer and the norbornene end-functionalized PEG chains displayed a reduced growth rate, crystallization kinetics, and crystallinity degree. A study of polymer chain architecture with linear-brush and star-brush PEGs of several molar masses has shown the constraints imposed by these architectures, which can reduce the crystallinity degree and overall crystallization rate.^15^ However, significant knowledge gaps should be addressed, such as the effect of the backbone topology and the role of the side chain conformation within the crystal.

**Figure 2:**
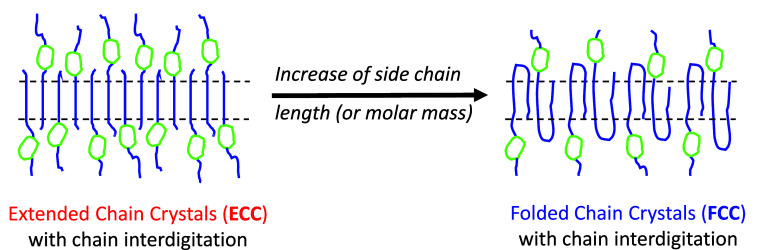
Simplified scheme showing the formation of interdigitated extended chain crystals and the possible formation of novel interdigitated FCCs by increasing the length of the PEG side chains of the cyclic comb polymers employed here (the chemical structure of the polymers employed in this work is shown in Figure 3).

**Figure 3:**
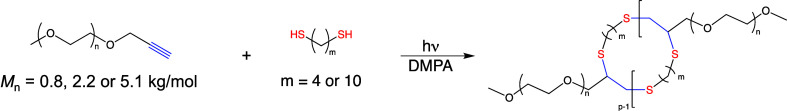
Chemical structure of dithiol–yne-based comb polymers with several alkanedithiol spacers (*m* = 4 and 10) and PEG side chains (0.8, 2.2, and 5.1 kg/mol) [for more details on the synthesis and chemical characterization, see ref (8)].

This work studies the self-assembly and crystallization of comb polymers with a dithiol–yne-based ring polymer backbone and PEG side chains (their chemical structure is shown in Figure 3). Two spacer lengths of the ring backbone were produced, and PEG side chains of several molar masses (from 0.8 to 5.1 kg/mol) were studied. This carefully designed system allows the investigation of the following issues for the first time: (a) the impact of having a ring backbone, which in principle could increase the constraints of the system; (b) the important variable of having different distances between neighboring PEG chains by varying the length of the spacer in the ring backbone, and (c) the length of PEG side chains, which in principle could produce extended or folded chain crystals (FCCs), see Figure 2. For comb polymers, interdigitated packing has been reported, with side chains adopting an extended conformation (interdigitated extended chain crystals, ECC). However, studies analyzing whether FCCs could be formed by increasing the length of the tethered PEG side chains have not been carried out for comb polymers, as far as the authors are aware. Figure 2 represents an idealized scheme of forming such interdigitated FCCs, which we have shown to be possible in this work, for the first time, according to evidence gathered by both SAXS and CryoTEM, as discussed below.

## 2 Materials and Methods

### 2.1 Materials

PEG homopolymers with one propargyl end group and several molar masses were synthesized. Then, comb polymers, constituted by a dithiol–yne-based ring polymer backbone and PEG side chains (see Figure 3), were obtained following the procedure reported before^8^ and summarized in the Supporting Information (SI). PEG homopolymers (without propargyl end group) were used for comparison purposes with number-averaged molecular weights of 0.8, 2.2, and 5.1 kg/mol (determined by GPC). The ring backbone’s size has been varied by employing alkylane dithiol spacers with four carbons (1,4-butanedithiol, BDT) and ten carbons (1,10-decanedithiol, DDT).

In total, nine samples have been investigated, see Table 1. The materials were dried overnight at 40 °C before performing the differential scanning calorimetry (DSC) studies.

**Table 1:** Materials Investigated and Their Corresponding Codes

molar mass of PEG chains^a^	linear homopolymers	BDT spacer in the ring backbone	DDT spacer in the ring backbone
0.8 kg/mol	0.8-LH	0.8-CP-BDT	0.8-CP-DDT
2.2 kg/mol	2.2-LH	2.2-CP-BDT	2.2-CP-DDT
5.1 kg/mol	5.1-LH	5.1-CP-BDT	5.1-CP-DDT

a Number-averaged molecular weight measured by GPC, the dispersity was lower than 1.15 for all PEG samples. LH refers to linear homopolymer and CP to comb polymer. BDT and DDT refer to the number of methylene groups in the spacer of the ring backbone.

The size of the cyclic comb, i.e., the *p* parameter in Figure 3, varies, obtaining a size distribution of cyclic comb polymers, as reflected in the polydispersity of the total molecular weight of the samples (see Table S1). However, in all cases, the distance between two neighboring PEG side chains is kept constant since this is defined by the spacer, having 4 or 10 methylene groups (Figure 3), depending on the sample. A detailed description of the synthesis and chemical characterization of the comb polymers used in this work can be found in a previous ref (8).

### 2.2 Differential Scanning Calorimetry

A PerkinElmer Pyris DSC 8000 was used with an Intracooler 2P. Indium and tin standards were employed as calibration standards. The sample mass was kept approximately constant at 5 mg. Ultrahigh purity nitrogen was employed as purge gas and the samples were encapsulated in standard PerkinElmer aluminum pans.

Nonisothermal crystallization tests were performed between −40 and 100 °C with a 20 °C/min scanning rate. Samples were left for 3 min at 30 °C above the peak melting temperature to create an isotropic melt state (i.e., its thermal history was erased). Then, they were cooled to −40 °C and kept for 1 min at this temperature to stabilize the calorimeter. Finally, they were once again heated to a temperature of 30 °C above the peak melting temperature.

The crystallinity percentage was calculated from the second DSC heating traces according to
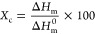
1where Δ*H*_m_ (J/g) is the fusion enthalpy and Δ*H*_m_^0^ (J/g) is the fusion enthalpy of a fully crystalline sample (214 J/g).^17^

DSC isothermal experiments were conducted to determine the overall crystallization kinetics. First, the minimum isothermal crystallization temperature (*T*_c,min_) is determined using the method proposed by Müller et al.^18,19^ Samples were cooled from the melt at 60 °C/min to selected *T*_c_ values and immediately heated up to 100 °C at 20 °C/min. This step was repeated, decreasing *T*_c_ values until, at a certain point, a melting endotherm was recorded in the heating scan. This temperature marks the lowest *T*_c_ that can be used, i.e., the lowest *T*_c_ temperature that does not show a melting endotherm in the subsequent heating. In this way, an appropriate *T*_c_ range in which the crystallization occurs entirely in an isothermal way was determined for each of the samples. Then, isothermal DSC experiments were conducted following Müller et al.^18,19^ The samples were first heated from 25 to 30 °C above their melting peak temperature at 20 °C/min. The samples were then rapidly cooled at 60 °C/min to a previously determined specific *T*_c_ value, and isothermal crystallization was performed until complete saturation (maximum of 30–40 min). Finally, the samples were heated from *T*_c_ to 100 °C at 20 °C/min to record their melting behavior.

### 2.3 Small-Angle and Wide-Angle X-ray Scattering

Small-angle X-ray scattering (SAXS) and wide-angle X-ray scattering (WAXS) measurements were simultaneously performed at the beamline BL11-NCD of the ALBA Synchrotron in Barcelona (Spain). Samples were contained inside DSC aluminum pans, and a Linkam THMS600 hot stage coupled to a liquid nitrogen cooling system was employed to cool and heat the samples at 20 °C/min. The same nonisothermal protocol used in the DSC tests was employed to obtain the SAXS/WAXS diffractograms during nonisothermal cooling and heating.

A 12.4 keV (λ = 1.03 Å) X-ray energy source was employed. The distance between sample and detector (ADSC Q315r detector, Poway, CA, USA, with a resolution of 3070 × 3070 pixels, pixel size of 102 μm^2^) was 6463 mm in the case of SAXS, with a tilt angle of 0°. Silver behenate was employed for calibration. Regarding the WAXS setup, a 132.6 mm sample to detector distance was used, with a tilt angle of 21.2°. The calibration was performed using Chromium(III) oxide (Rayonix LX255-HS detector, Evanston, IL, USA, with a 1920 × 5760 pixels resolution, pixel size of 44 μm^2^). Plots of the scattering intensity as a function of the scattering vector were obtained for both SAXS and WAXS data, where the scattering vector is defined as *q* = 4πsin θλ^–1^, and λ is the X-ray wavelength, and 2θ the scattering angle.

### 2.4 Polarized Light Optical Microscopy

An Olympus BX51 polarized light optical microscope was employed to observe the samples’ morphology and determine their superstructural (spherulitic or axialitic) growth rate. The morphological changes that occurred while cooling and heating the samples at 20 °C/min were followed, employing a Linkam THMS600 hot stage with liquid nitrogen for temperature control. An Olympus SC50 camera was used for image recording.

First, the samples are prepared in a glass slide with a glass coverslip, in which samples are melted. For nonisothermal tests, all samples were first heated to 80 °C and kept at this temperature for 3 min to ensure complete melting. Then, samples were cooled to 0 °C and then heated to 80 °C at 20 °C/min. All morphological changes that occurred were recorded as polarized light optical microscopy (PLOM) images in which sample crystallization and melting can be followed.

The spherulitic growth rate (*G*) was measured during isothermal experiments. The samples were prepared by heating a small amount of material between two glass slides to 80 °C and keeping it at that temperature for 3 min to ensure an isotropic melt state was reached. Then, the samples were cooled at 50 °C/min to a crystallization temperature where axialites or spherulites appeared. The spherulitic/axialitic growth rate at a specific temperature was isothermally followed as a function of time, recording morphological changes by taking micrographs. This procedure was repeated several times for different temperatures. In each of the temperatures, the radius of spherulites was measured and plotted as a function of time, and straight lines were always obtained where the slope gives the spherulitic growth rate (*G*).^20^

### 2.5 Transmission Electron Microscopy

The samples were microtomed employing Leica EM UC7 cryo-ultramicrotome at −120 °C with a Diatome 25° diamond knife. Sections with an approximate thickness of 70 nm were deposited onto a 300 mesh lacey carbon grid. The sections were dried under vacuum to prevent moisture accumulation and possible alterations to the original microstructure. Then, the grids were exposed to RuO_4_ vapor for 10 min, and finally, they were transferred to the microscope.

CryoTEM experiments were carried out on a Schottky field-emission cryo-electron microscope (model JEOL JEM-3200FSC), with an accelerating voltage of 300 kV. During the whole process, the samples were kept at −187 °C. Micrographs were captured using the bright field mode, with a zero energy loss omega-type filter and a slit width of 20 eV. The images were acquired with a Gatan Ultrascan 4000 CCD camera employing the Gatan Digital Micrograph software.

## 3 Results and Discussion

### 3.1 Non-Isothermal Crystallization

The structure of the comb materials after crystallizing under nonisothermal conditions was investigated by WAXS and SAXS. The thermal properties were analyzed employing DSC. A detailed description of these studies is included in the Supporting Information. All the homopolymers and comb materials can crystallize under the conditions studied.

WAXS analysis showed almost identical WAXS patterns for PEG homopolymers and comb polymers, with no shift in diffraction peaks. This implies that only PEG side chains crystallize, and the comb backbone is rejected to the amorphous phase.

**Figure 4:**
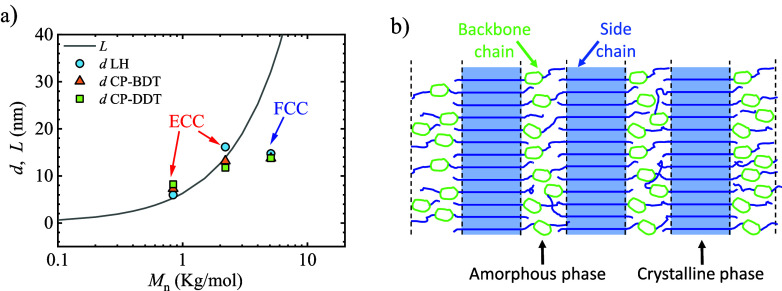
(a) Long period, *d*, obtained from SAXS measurements as a function of molar mass (data points). The solid line represents the length of the chains in an extended conformation inside the crystals, *L*. ECCs are formed in the case of 0.8 and 2.2 kg/mol PEO samples, and FCCs in the case of 5.2 kg/mol sample. (b) Simplified scheme showing the structure of comb polymers with PEG interdigitated side chains forming the crystalline phase and the cyclic comb backbone and the chain segments close to it constituting the amorphous phase.

Since PEG samples with low molar mass can form ECCs or FCCs, SAXS studies have been performed to ascertain the conformation of the chain in the studied samples. The long period has been estimated from the *q* value corresponding to the maximum from SAXS patterns, and the values are compared with the estimated length of extended chain crystals, considering that they adopt a 7/2 helix crystalline structure, see Figure 4a.

The homopolymers and comb polymers with 0.8 and 2.2 kg/mol molar masses have a similar long period to the extended chain length; thus, ECCs are formed. A simplified scheme of the structure of the material is provided in Figure 4b, showing that the side chains form the crystal and that the cyclic comb backbone and the chain segments close to the comb constitute the amorphous phase. For samples with 5.1 kg/mol, the long period obtained from SAXS is much smaller than the estimated length of extended chains; thus, FCCs are formed for both the homopolymer and the comb polymers (as indicated in Figure 2). It is relevant to highlight that although chain interdigitation is usually reported for comb systems with chains in an extended conformation (as the side chains are usually quite short), PEG side chains that are long enough (i.e., 5.1 kg/mol) are able to fold. A more detailed discussion of these nonisothermal results is included in the Supporting Information.

### 3.2 Isothermal Crystallization

#### 3.2.1 Superstructural Morphology Investigated by PLOM

**Figure 5:**
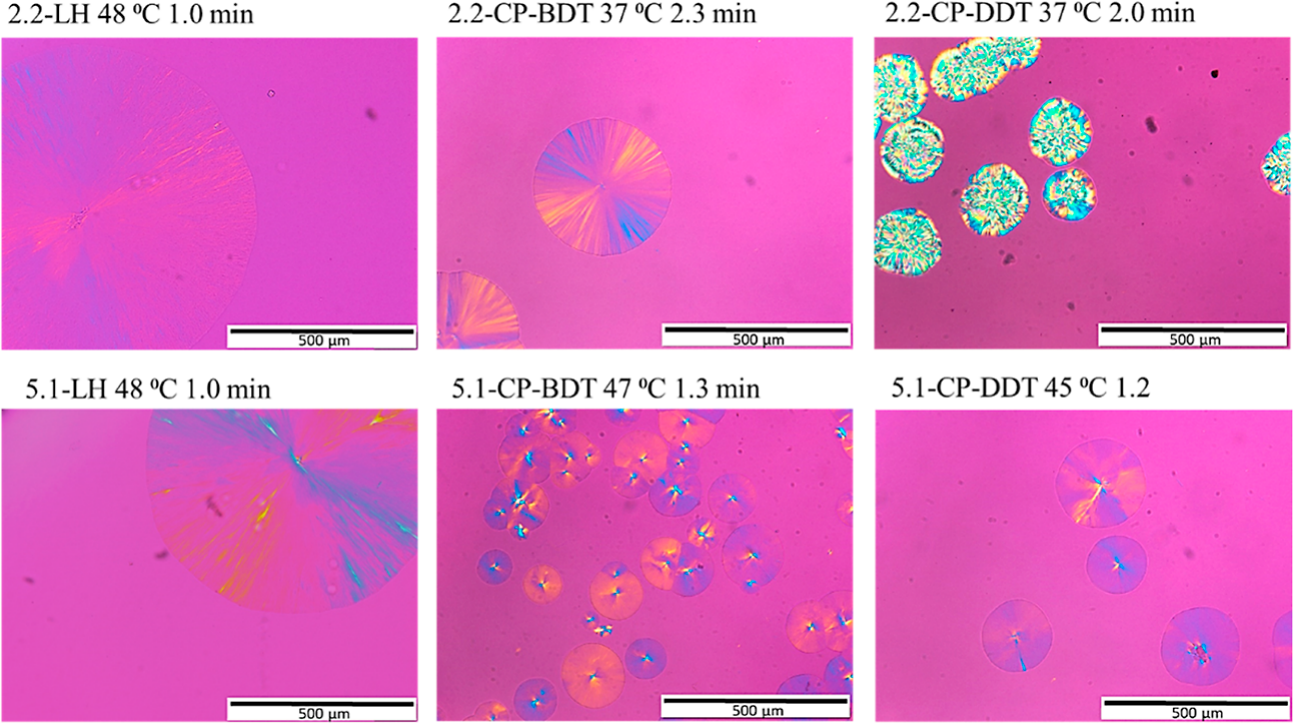
PLOM images obtained after cooling down from the appropriate melting temperature at 50 °C/min to the selected crystallization temperature.

The morphology of the materials was investigated by PLOM. The samples were crystallized isothermally after cooling down from the appropriate melting temperature at 50 °C/min. Figure 5 shows typical micrographs taken during the initial steps of the superstructural growth to analyze the morphology of PEG 2.2 and 5.1 kg/mol linear homopolymers and the corresponding comb polymers. It was impossible to follow the isothermal crystallization of the PEG homopolymer and comb polymers with 0.8 kg/mol because of the proximity of the melting and crystallization temperatures. Thus, upon cooling from the melt at 50 °C/min down to *T*_c_, the material always started to crystallize before reaching the isothermal crystallization temperature.

The evolution of the superstructural growth as a function of time at selected *T*_c_ values is shown in the Supporting Information; see Figures S6–S11. The images in Figure 5 were taken at different *T*_c_ values since the homopolymers need much lower supercoolings than comb polymers to crystallize. The studied samples crystallized, apparently forming crystalline superstructures that, in most cases, correspond to spherulites. It is worth noting that even with the constraining effect of having one chain end linked to the ring backbone, the material can self-assemble at the microscopic level to form three-dimensional spherulitic superstructures (i.e., spherulites) and, in some cases, with the typical Maltese cross extinction patterns characteristic of the PEG homopolymers. The morphology of the spherulites varies for the comb polymers.

It seems that linear polymers exhibit a lower nucleation density since only one big spherulite is observed for them at low crystallization times, whereas for comb polymers, several spherulites appear. However, the samples in Figure 5 are compared at different *T*_c_ values and times. A proper nucleation study would have to be performed to reach a more definite conclusion in a wide range of *T*_c_ and times values, but this is outside the scope of the present work.

The change in spherulitic morphology for the comb polymers is quite peculiar. The spherulites obtained for the two linear PEG samples and the 2.2-CP-BDT show the typical Maltese cross-pattern with a negative sign. This pattern is not so clear for the 5.1 kg/mol comb polymers. The 2.2-CP-DDT sample shows a very peculiar superstructural morphology, where a banding extinction pattern can sometimes be observed.

#### 3.2.2 Structure and Morphology of the Isothermally Crystallized PEG Chains Analyzed by WAXS, SAXS, and TEM: Understanding the Self-Assembly Induced by Chain Topology

**Figure 6:**
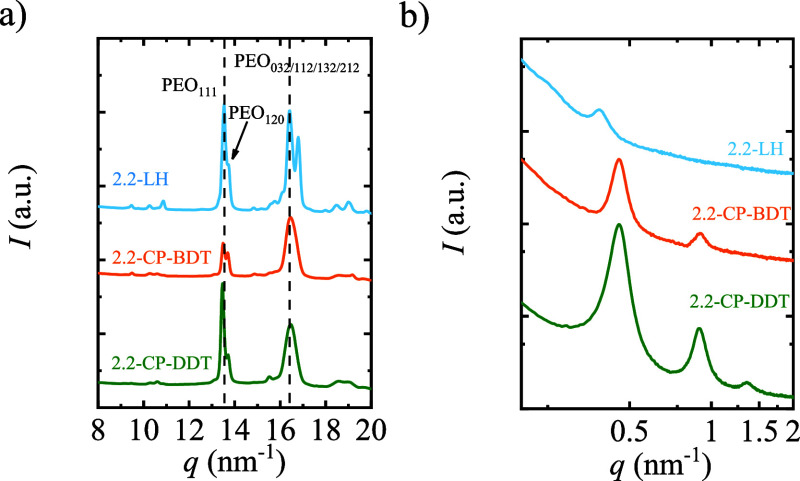
a) WAXS and (b) SAXS patterns of 2.2-LH, 2.2-CP-BDT and 2.2-CP-DDT after isothermally crystallizing the sample at 38 °C for 60 min.

WAXS and SAXS experiments were performed to analyze the crystalline structure of isothermally crystallized samples. Figure 6 shows the WAXS and SAXS patterns for linear and comb polymers with 2.2 kg/mol PEG chains crystallized at 38 °C samples.

The WAXS patterns are similar to those obtained under nonisothermal conditions and corroborate that PEG crystallizes with a monoclinic unit cell in all cases; thus, only PEG side chains crystallize in the comb polymers employed here. The main peak around 13.6 nm^–1^ corresponds to the (111) plane, and the small peak near 13.8 nm^–1^ corresponds to the (120) plane. The signal around 16.5 nm^–1^ results from the overlap of the scattering reflections from the (032), (112), (132), and (212) planes.^21,22^

The long period (*d*) is determined by the value of the scattering vector (*q*) of the maxima of the highest intensity SAXS peaks through the equation (*d* = 2π/*q*), and corresponds to the sum of the crystalline lamellar thickness (*l*_c_) and amorphous lamellar thickness (*l*_a_).^23,24^ Combining *d* determined by SAXS (Figure 6b) and the crystallinity degree determined by WAXS (Figure 6a), it is possible to have a good approximation of the average values of *l*_c_ and *l*_a_, shown in Table 2 for both homopolymers and comb polymers. The crystallinity degree obtained by WAXS, long period, and calculated *l*_c_ and *l*_a_ are shown in Table S3.

**Table 2:** Long Period (*d*), Crystalline Lamellar Thickness (*l*_c_), and Amorphous Layer Thickness (*l*_a_) Measured by SAXS (with Crystallinity Degree Estimated by WAXS) and TEM and the Calculated Extended Chain Length Considering That the PEG Chains Adopt a Helical 7/2 Conformation^24^

sample	*d*_SAXS_ (nm)	*l*_c,SAXS_ (nm)	*l*_a,SAXS_ (nm)	*d*_TEM_ (nm)	*l*_c,TEM_ (nm)	*l*_a,TEM_ (nm)	*L* (nm)
2.2-LH	16.0	12.2	3.8	16 ± 6	12 ± 5	4 ± 6	13.9
2.2-CP-BDT	13.8	9.2	4.6	14 ± 1	10 ± 1	4 ± 1	13.9
2.2-CP-DDT	13.8	8.5	5.3	15 ± 1	8 ± 1	7 ± 1	13.9
5.1-LH	16.4	12.7	3.7	17 ± 4	13 ± 4	4 ± 4	32.2
5.1-CP-BDT	23.5	13.6	9.9	33 ± 10	20 ± 8	13 ± 10	32.2
5.1-CP-DDT	12.4	8.1	4.3	15 ± 2	11 ± 2	4 ± 2	32.2

**Figure 7:**
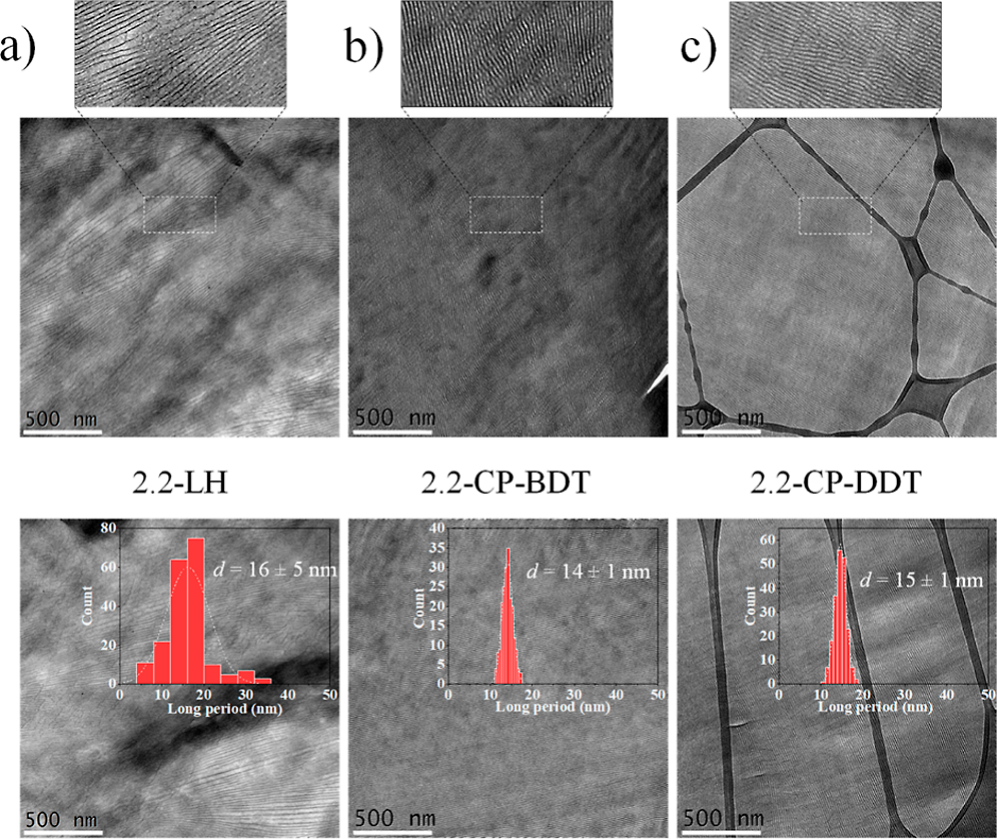
TEM micrographs for (a) 2.2-LH PEG, (b) 2.2-CP-BDT, and (c) 2.2-CP-DDT after isothermally crystallizing the materials at 38 °C.

While for the PEG homopolymer, a broad SAXS peak centered at *q* = 0.39 nm^–1^ is observed, for the comb polymers that contain tethered PEG chains of identical lengths, better-defined SAXS peaks are observed centered at *q* = 0.46 nm^–1^, Figure 6b. Besides, second-, and second-/third-order reflections centered at *q* = 0.91 and 1.35 nm^–1^ are clearly observed for 2.2-CP-BDT and 2.2-CP-DDT. Better-defined reflections and the presence of higher orders confirm that the self-assembly of crystalline lamellar stacks for the comb-like cyclic polymers exhibits higher ordering and smaller domain size distribution than that of the PEG homopolymer.^23^ The SAXS scattering peaks are related to a lamellar morphology,^25−28^ which can be clearly observed in the transmission electron microscopy (TEM) images presented in Figure 7.

RuO_4_ staining of semicrystalline PEG samples leads to increased electron contrast on the amorphous phase, which allows higher coordination density of Ru nuclei with oxygen atoms from the PEG backbone.^27,28^ Therefore, whiter regions seen on the TEM images of Figure 7 represent crystalline lamellae, while darker regions correspond to the amorphous interlamellar domains. The long-period (*d*_TEM_), crystalline lamellar thickness (*l*_c,TEM_), and amorphous lamellar thickness (*l*_a,TEM_ = *d*_TEM_ – *l*_c,TEM_), calculated with the measurement of over 200 interlamellar distances and crystalline lamellar thicknesses, respectively, are shown in Table 2. Besides, histograms regarding *l*_c,TEM_ are shown in Figure 7. The *l*_c,TEM_ distribution is broad for the 2.2-LH PEG sample, in contrast to the narrower crystalline lamellar thickness distribution of the 2.2-CP-BDT and 2.2-CP-DDT comb-like polymers.

**Figure 8:**
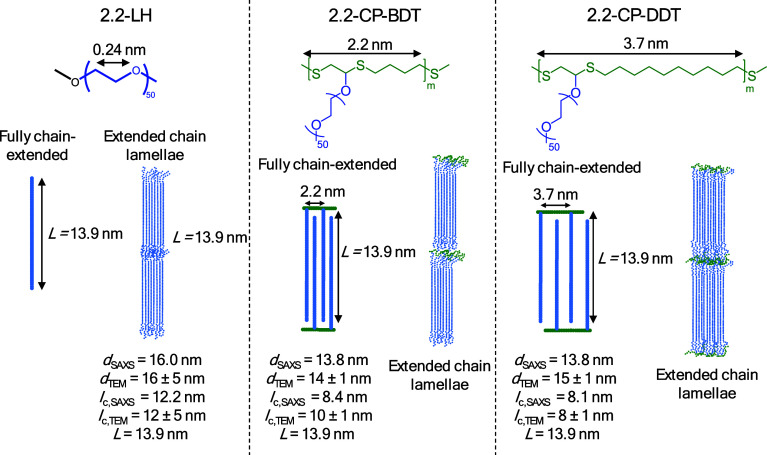
Proposed model for 2.2-LH PEG, 2.2-CP-BDT, and 2.2-CP-DDT, which form extended-chain lamellae. The long period (from SAXS), crystalline lamellar thickness (from TEM), and the extended chain length (calculated considering a 7/2 helical conformation and denoted *L*) are shown in the figure.

As PEG can crystallize into ECC or FCC lamellar crystals,^24,27^ a model regarding PEG and its cyclic comb-like polymers can be achieved using (1) unit cell parameters for PEG monoclinic structure; (2) the chain length of PEG chain-extended crystals according to 7/2 helical conformation,^24^Table 2, and (3) the information acquired by SAXS, WAXS, and TEM,^23^Figure 8. The molar mass distribution of both PEG homopolymers and comb-like polymers is discussed in a previous publication.^8^ The PEG used in this study is not monodisperse. Even if the dispersity for the homopolymer PEG samples (*D̵* < 1.2) is relatively low, the molar mass distribution is wide enough to produce an inevitable broadening of the lamellar thickness distribution.

As reported in the literature, PEG homopolymers with a molar mass of 2 kg/mol crystallize into ECC lamellar crystals.^29,30^ Our SAXS and TEM results support this observation from the literature for the nonisothermally crystallized samples, as discussed above, but also for the homopolymer samples shown in Figures 6b and 7a that have been isothermally crystallized. According to Figure 8a, the chain length of chain-extended PEG crystals (*L*) in the crystallographic registry (*M*_n_ = 2.2 kg/mol) is approximately 13.9 nm, and therefore very similar to the values of *l*_c_ estimated by SAXS, Table 2. The average crystalline lamellar thickness obtained by TEM also agrees with such chain extended conformation, with an average value of 12 ± 5 nm. The observation by TEM of a minority of thicker lamellas (>25 nm) can be explained by partial chain interdigitation or formation of bilayers,^31^ see Figure S12, and/or the dispersity of the molar mass distribution. The lamellar thickness determination on the TEM micrographs was performed, supposing all lamellae are seen edge-on using the open-source ImageJ software.

As confirmed by SAXS (Figure 6b) and TEM (Figure 7), the lamellae are much better defined in terms of size distribution and packing on both comb-like polymers, when compared to PEG homopolymer, which gives rise to their better-defined reflections and second-/third-order reflections observed in SAXS plots, Figure 6. We hypothesize that this is due to a crystallization-induced self-assembly effect promoted by the comb-like architecture of the cyclic block copolymers. This hypothesis is strengthened by the fact that no evidence supports microphase-separation in the amorphous phase and the melt for the comb polymers. Therefore, upon crystallization, the polythioether backbone is excluded from the crystalline phase into the interlamellar amorphous region, together with PEG chain-ends, and PEG segments covalently connected and spatially close to the polythioether backbone. However, such an amorphous polythioether backbone is connected to several PEG chains. Statistically, for PEG homopolymers, it is likely that a single chain is present in two distinct amorphous layers; in contrast, for PEG comb-like polymers, several PEG chains must necessarily share the same interlamellar amorphous region due to covalent connection with the polythioether backbone. This leads to better ordering and thinner lamellar thickness distributions. PEG homopolymer chains have no interchain spatial constraints and have higher freedom in mobility and spatial arrangement; the obtained morphologies after crystallization are more disordered and possess broader lamellar thickness distribution.

Besides, such a polythioether backbone increases the amorphous lamellar thickness of the comb-like polymers, *l*_a,SAXS_ = 4.6 nm for the 2.2-CP-BDT and 5.3 nm for the 2.2-CP-DDT, compared with *l*_a,SAXS_ = 3.8 nm for the 2.2-LH PEG. The tethering also implies that it is statistically likely that PEG side chains of a single macromolecule (that comprises one ring backbone with several PEG side chains) are participating in different crystalline lamellae (i.e., by extended chain interdigitation); therefore, inputting an intermediate amorphous phase in between the crystalline lamellae formed partly by the polythioether backbone. In the TEM images, Figure 7, and also the enlarged images presented in Figure S12 of the Supporting Information, this effect is visualized by the presence of several branched, amorphous lamellae that interconnect three or more crystalline lamellae for the cyclic comb-like polymers (as indicated by the drawn white circles in the images). Such amorphous lamellae that interconnect three or more crystalline lamellae are scarce in the 2.2-LH PEG. Since the polythioether is not present in the PEG homopolymers, the crystalline domains may interconnect with small defects poorly visible by TEM and in any direction with respect to adjacent lamellas, leading to the broader lamellar thickness distribution.

Since the distance between neighboring PEG side chains in 2.2-CP-DDT is less than 4 nm, see Figure 8, and the molecular weight of the PEG side chains is only 2.2 kg/mol, chain interdigitation and the formation of ECC crystals is favored. Also, a twist of the polythioether ring backbone in the amorphous regions may occur to allow the packing of PEG side chains into crystalline lamellae (see Figure S12). In comparison with PEG homopolymer, *l*_a_ increases and *l*_c_ decreases in the comb polymers. Moreover, such effect is enhanced by the increase in the length between side chains, see Figure 8, as *l*_a,2.2-CP-DDT_ > *l*_a,2.2-CP-BDT_, see Table 2.

**Figure 9:**
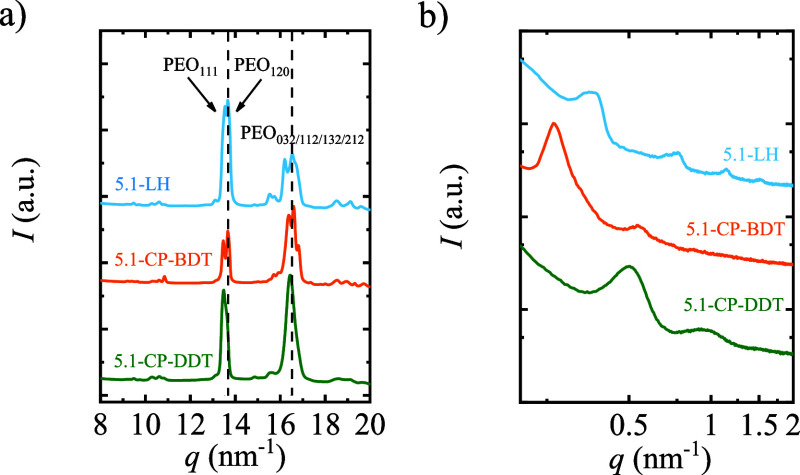
(a) WAXS and (b) SAXS patterns of 5.1-LH, 5.1-CP-BDT and 5.1-CP-DDT after crystallizing the sample at 46 °C.

**Figure 10:**
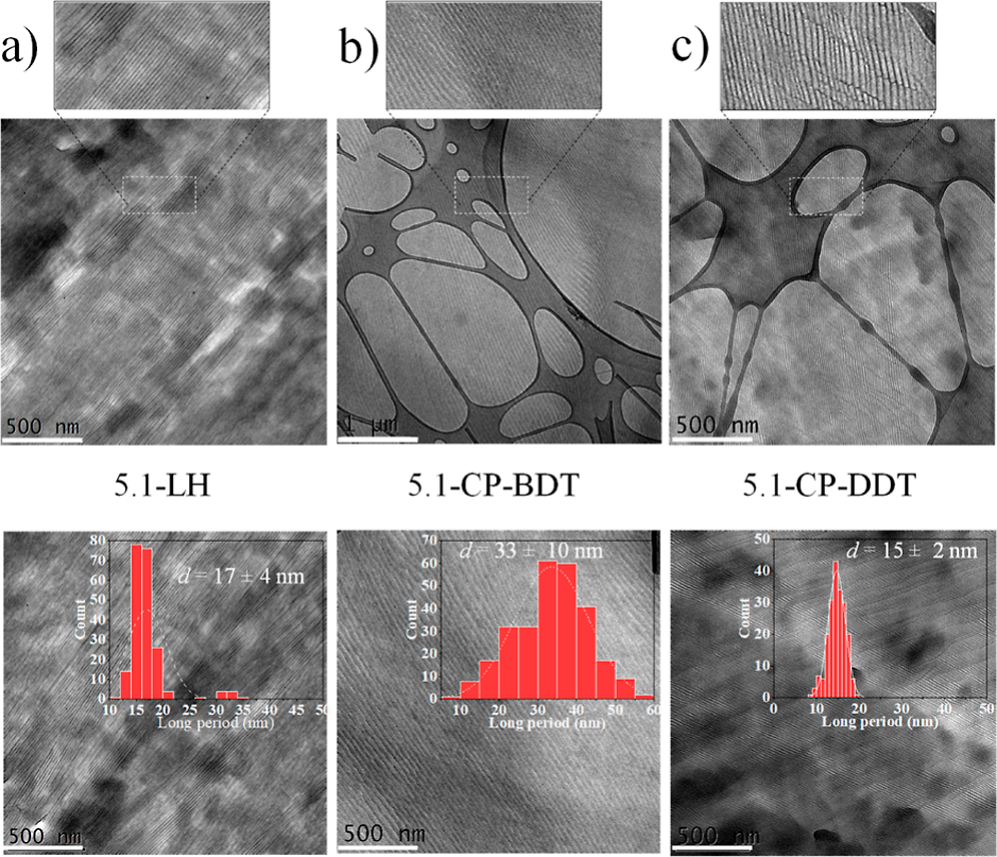
TEM micrographs for (a) 5.1-LH PEG, (b) 5.1-CP-BDT, and (c) 5.1-CP-DDT after isothermally crystallizing the materials at 46 °C.

In Figure 9, the WAXS and SAXS patterns of the 5.1 kg/mol samples are shown. WAXS experiments confirmed that a monoclinic unit cell is formed, as could be expected. SAXS measurements for PEG 5.1-LH PEG show a scattering peak centered at *q* = 0.38 nm^–1^, and second-, third-, and fourth order reflections centered at *q* = 0.76, 1.14, and 1.51 nm^–1^, respectively, suggesting a highly ordered microphase separated morphology. This is a consequence of the crystallization of PEG into well-defined crystalline lamellae separated by also well-defined amorphous interlamellar regions, as verified by TEM, see Figure 10.

**Figure 11:**
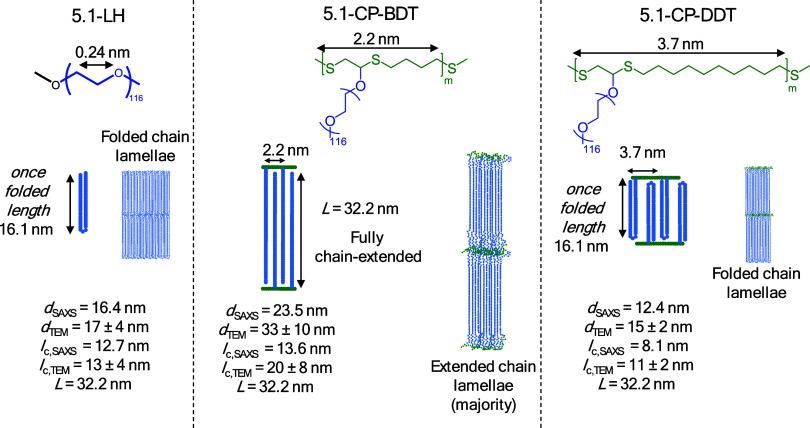
Proposed model for 5.1-LH PEG, 5.1-CP-BDT, and 5.1-CP-DDT. The long period (from SAXS), crystalline lamellar thickness (from TEM), and the extended chain length (calculated considering a helical conformation) are shown in the figure.

As previously mentioned, using the same analysis of SAXS and TEM results associated with the crystallization-induced chain-folding theory, it is possible to infer that 5.1-LH crystallizes into FCC lamellar crystals, with approximately one fold per chain, see Figure 11. This can be assumed since both *l*_c,SAXS_ + *l*_a,SAXS_ = 16.4 and *l*_c,TEM_ + *l*_a,TEM_ = 17 ± 4 nm, determined by SAXS and TEM, respectively, are close to the length of a single-folded PEG 5.1 kg/mol chain (16.1 nm), considering such as half the length of a PEG 5.1 kg/mol ECC (32.2 nm). Besides, *l*_c,TEM_ and *l*_c,SAXS_ for the 5.1-LH PEG are 13 ± 4 and 12.7 nm, respectively, which is similar to the estimated length of a single-folded PEG 5.1 kg/mol chain (16.1 nm). Apparently, crystallization-induced chain-folding provides better-lamellar stacking for the PEG 5.1 kg/mol homopolymer, when compared to the ECC structure for PEG 2.2 kg/mol homopolymer, as judged by the better resolved SAXS pattern.

Regarding the cyclic comb-like polymer with the shorter spacer 5.1-CP-BDT, three peaks are observed in the SAXS profiles, see Figure 9, the highest intensity one is centered at 0.27 nm^–1^, and the second- and third reflections centered at 0.54 and 0.56 nm^–1^, respectively. The long period determined by SAXS of 23.5 nm and TEM of 33 ± 10 nm is far larger than the chain length of a once-folded PEG side-chain, which is 16.1 nm. Therefore, it is reasonable to infer that upon isothermal crystallization, the 5.1-CP-BDT comb-like polymer achieves a crystalline structure with a majority of ECC lamellar crystals, which have a theoretical chain length of 32.2 nm. Chain interdigitation is expected in the formed ECC crystals (see Figure S13). A smaller amount of chains could also undergo chain folding according to the histograms presented in Figure 10 and averages reported in Figure 11 and Table 2. Given the folded-chain lamellar structure of the 5.1 kg/mol PEG homopolymer, such a difference in the lamellar structure of the comb polymer can only be related to the tethering of PEG on the amorphous polythioether backbone. It is likely that the presence of relatively long PEG chains (32.2 nm) intercalated by a short spacer of 2.2 nm length in a single macromolecule, provided by the comb-like architecture of the cyclic 5.1-CP-BDT, leads to steric hindrances for chain folding. This is true, especially considering that the unit cell parameters for PEG crystals (*a* = 0.805 nm, *b* = 1.304 nm, and *c* = 1.948 nm) are within the size range of the side chain spacer (i.e., 2.2 nm) of 5.1-CP-BDT, see Figure 11. Interestingly, in the case of nonisothermal crystallization at 20 °C/min, the same 5.1-CP-BDT sample did form FCC lamellae, probably because of kinetic reasons.

In the case of the 5.1 kg/mol PEG comb-like polymer with the longer spacer 5.1-CP-DDT (3.7 nm), the steric hindrance between neighboring PEG side chains is lowered, and both long-period, determined by SAXS and TEM, respectively, are closer to the length of a chain-folded lamella with once folded PEG side-chains of 16.1 nm (Figure 11), than to the length of an extended chain lamella (32.2 nm). This fact supports the predominance of FCC lamellar crystals for the 5.1-CP-DDT sample, as shown in Figure 11. The formation of interdigitated folded chain crystals for side chains in comb polymers has not been reported for comb polymers with PEG side chains, as far as the authors are aware. The *l*_c,TEM_/*l*_c,WAXS_ for the 5.1-CP-DDT are smaller than that of the 5.1 kg/mol homopolymer, while *l*_a_ values are higher, due to the presence of the polythioether backbone that leads to interdigitation and backbone-twisting, as previously discussed. The same effect of increased ordering and a thinner lamellar thickness distribution provided by the tethering of PEG side-chains into an amorphous polythioether backbone is verified with the 5.1-CP-DDTsample, as shown in Figure 10, comparing with the PEG 5.1-LH PEG. The zoomed TEM image of the 5.1-CP-DDT sample, see Figure S14, seems to indicate that interlamellar amorphous regions may be interconnected into a branched “ladder-like” structure. Such structure could be caused by the participation of a single cyclic macromolecule into two or more crystalline lamellas, which gives rise to interconnecting amorphous phases due to the amorphous polythioether backbone.

#### 3.2.3 Spherulitic Growth Rate by PLOM: Crystal Growth Only Kinetics

The spherulitic growth rates under isothermal conditions were studied by PLOM. The samples were melted to reach an isotropic melt state and then cooled down to the selected crystallization temperature at 50 °C/min. The growth rate was determined by measuring the radius of the spherulites as a function of time, which always resulted in straight lines whose slope gives the values of the growth rates, *G*. The spherulitic growth rate of polymeric materials is characterized by a bell-shaped curve when plotted as a function of crystallization temperature (in the temperature window between the glass transition temperature, *T*_g_ and *T*_m_). This trend results from the competition of chain diffusion (at low *T*_c_ values, in the left-hand side region of the curve, where melt viscosity rapidly increases as *T*_c_ decreases) and secondary nucleation (at high *T*_c_ values, where the secondary nucleation probability decreases as chain mobility rapidly increases as temperature increases).

**Figure 12:**
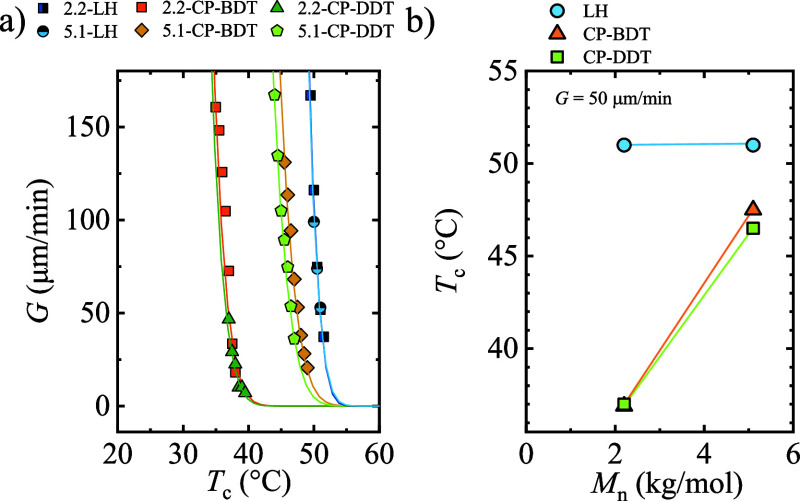
(a) Spherulitic growth rate as a function of isothermal crystallization and (b) crystallization temperature to reach a growth rate of 50 μm/min. The straight lines are shown to guide the eye.

Figure 12 shows the *G* values as a function of *T*_c_ for the samples investigated in this study. The PEG sample with 0.8 kg/mol could not be studied due to the high nucleation density. For PEG 2.2 and 5.1 kg/mol samples, *G* values sharply increase when *T*_c_ decreases; thus, only part of the right-hand side of the bell shape curve can be observed, in which secondary nucleation is the limiting step. Figure 12a shows that PEG homopolymers need smaller supercoolings to crystallize than comb polymers. Both PEG homopolymer samples exhibit identical growth rate values as a function of *T*_c_, regardless of the molecular weight. However, in the case of the comb polymers, the 5.1-CP samples need much smaller supercoolings to crystallize than the 2.2-CP samples.

To compare the behavior of the samples, a constant value of *G* can be considered, and the corresponding *T*_c_ can be compared. Figure 12b shows the *T*_c_ values for *G* values equal to 50 μm/min. The results indicate that the homopolymers show the highest *T*_c_ values, meaning that lower supercoolings are needed to reach a *G* value of 50 μm/min value. Interestingly, PEG 2.2 and 5.1 kg/mol need the same supercooling (same *T*_c_) to reach a 50 μm/min rate. Godovsky et al. investigated several PEG fractions covering low molar mass samples.^3^ They observed that there was a minimum in the spherulitic growth rate measured by DSC at around 4 kg/mol, which was related to the transition from ECC to FCC lamellar crystals. So, in that temperature region, the samples have a similar rate. Kovacs et al. analyzed the spherulitic growth rate of PEG samples with 2 and 4 kg/mol molecular weights and found that the growth rate does not differ much when measured isothermally around 50 °C.^33^

Comb-like polymers show a reduction in the *T*_c_, equivalent to an increase in the supercooling in Figure 12b. The PEG 2.2 kg/mol comb polymer needs the highest supercooling to reach a 50 μm/min rate in Figure 12b, with a dramatic reduction in the *T*_c_. Comb polymers with 2.2 kg/mol PEG side chains need a similar supercooling to crystallize independent of the length of the alkane dithiol spacer in the backbone ring. However, for PEG side chains of 5.1 kg/mol, increasing the alkane dithiol spacer slightly increases the supercooling needed for crystallization. This small difference may be related to the fact that 5.1-CP-BDT forms mostly ECC lamellae, while 5.1-CP-DDT tends to produce FCC lamellar crystals, according to Figures 10 and 11.

The observed increase in the supercooling for comb polymers arises from the constraints imposed by the covalent link between one extreme of the PEG chains and the ring backbone. Similar increases in the supercooling have been observed for brush PEG with poly(norbornene) backbones.^14^ Studies carried out with linear, star, and brush PEG have revealed that increasing the branching degree restricts the mobility of the chains, depressing the overall crystallization rate.^15^

Considering previous works carried out with cyclic and linear PEG chains of 2 kg/mol studying spherulitic growth rate^34^ and the comb polymers studied here with the same molar mass, the results indicate that cyclic chains diffuse faster than the linear analogues (as a consequence of their lack of chain ends and more collapsed coil conformation, see ref (35). and references therein). In contrast, comb polymers diffuse slower due to the chain tethering effect.

#### 3.2.4 Overall Crystallization Rate by DSC: Primary Nucleation and Growth Kinetics

**Figure 13:**
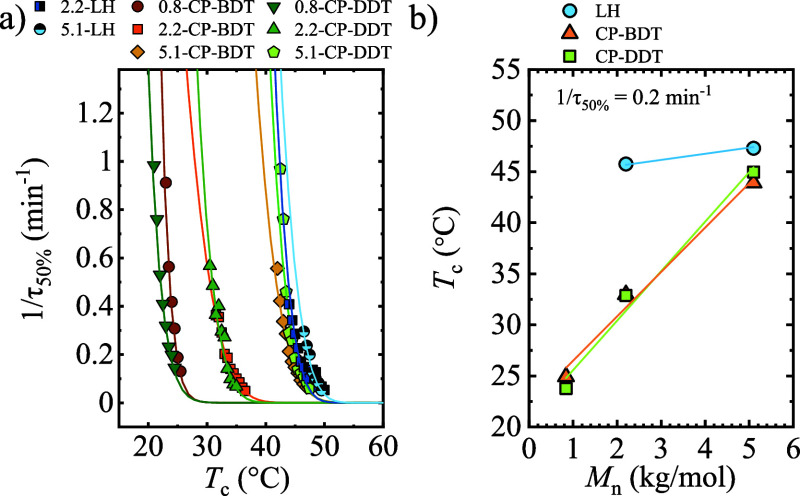
(a) Overall crystallization rate as a function of *T*_c_. The lines correspond to the LH fit. (b) Crystallization temperature required to reach 1/τ_50%_ = 0.2 min^–1^ rate. The straight lines are shown to guide the eye.

The overall crystallization kinetics that comprises nucleation and growth has been studied by DSC. The experimentally determined overall crystallization rate, 1/τ_50%_, as a function of *T*_c_ is shown in Figure 13a, which comprises both nucleation and growth. This parameter is defined as the inverse of the half crystallization time, which is the time needed to reach a 50% of the relative crystallinity that the polymer can reach.^18,19^ The materials show the expected behavior, with an increase in the overall crystallization rate as *T*_c_ decreases. The overall crystallization rate also tends to follow a bell shape trend when plotted versus *T*_c_ in a very large temperature range in between *T*_g_ and *T*_m_. The experimentally available *T*_c_ values are within the right-hand side of the bell shape curve, where the behavior is dominated by combined primary and secondary nucleation.

A crystallization rate of 0.2 s^–1^ is considered to compare the behavior of the studied samples. The linear polymers have the highest *T*_c_ (equivalent to lowest supercooling), as could be expected. In the case of comb polymers, the PEG 5.1 kg/mol samples show a slight shift to lower *T*_c_ values, whereas 2.2 kg/mol samples display a more significant shift of around 10 °C. The lowest *T*_c_ values are shown by the PEG 0.8 kg/mol comb-like polymers. The results indicate that PEG chains linked to the backbone have restricted mobility in comparison with the linear polymers in which the two chain ends are free to move, and this restriction reduces the crystallization rate. This is in line with previous studies carried out with star and brush PEG samples in which a reduction of crystallization rate with the branching degree has been reported.^15^

Comparing Figure 13a, where the results of the overall crystallization rate are presented (which include both primary nucleation and growth) with the trend obtained by PLOM where only growth rate was determined (Figure 12), it is possible to determine in a qualitative way which is the dominant factor in the overall crystallization rate (obtained by DSC). In general, the trends are similar in the three processes, except for PEG 5.1 kg/mol comb polymers. In this case, the materials show a significantly reduced *T*_c_ in the spherulitic growth rate (increased supercooling), whereas this effect is more subtle in the overall crystallization rate. This implies that overall crystallization rate is dominated by the primary nucleation rate for 5.1 kg/mol comb polymers.

The experimental data obtained from the isothermal DSC experiments has been fitted to the Avrami theory.^32,36,37^ The theory can usually predict the overall primary crystallization behavior below 50% of the relative crystallinity of the polymer (i.e., before spherulites impinged on one another).^32,36,37^ The Avrami theory can be expressed by the following modified equation^18,19^
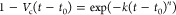
2where *V*_c_ is the relative volumetric transformed fraction, *n* is the Avrami index, *t* is the time corresponding to the isothermal experiment, *t*_0_ is the induction time, and *k* is the overall crystallization rate constant.^18,19^ An example of the fitting is given in the Supporting Information, Figure S15. The experimentally obtained heat flow and the one from the Avrami theory show a good correlation, which implies that Avrami theory is suitable to describe the primary crystallization of these materials. The data of the fittings for all the materials are displayed in the Supporting Information, Tables S4–S6.

**Figure 14:**
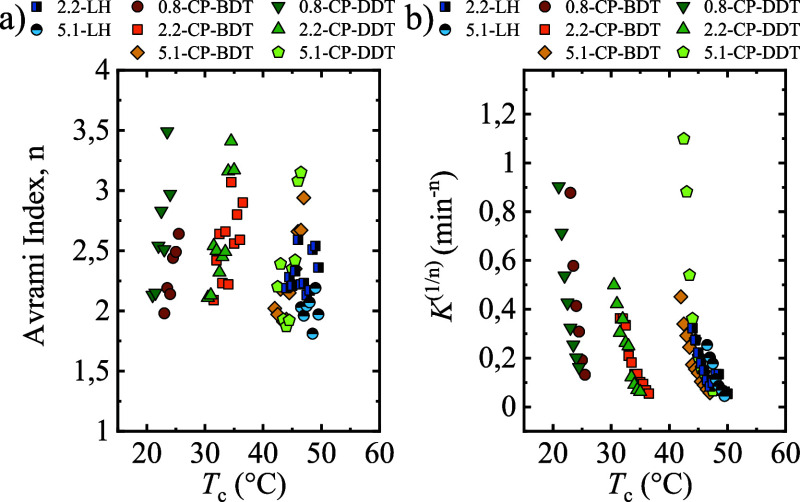
(a) Avrami index and (b) overall crystallization rate constant as a function of the isothermal crystallization temperature.

Figure 14a shows the Avrami index as a function of crystallization temperature. The homopolymers display Avrami index values between 2 and 2.6, which can be approximated as values that increase with *T*_c_ from 2 to approximately 3. In the present case, *n* = 2 can be interpreted as arising from instantaneously nucleated axialites, whereas *n* = 3 can correspond to sporadically nucleated axialites or instantaneously nucleated spherulites.^18,19,32^ Comb-like polymers show a broader Avrami index values, from 2 to approximately 4 (i.e., 3.5). An Avrami index of *n* = 4 in polymers corresponds to sporadically nucleated spherulites. The increase of Avrami index when increasing the isothermal crystallization temperature is usually due to a change in nucleation mechanism, from instantaneous to sporadic.^18,19,32^

The overall crystallization rate constant (normalized by elevating it to the power 1/*n*, so that the units are expressed in min^–1^^18,19^) is shown in Figure 14b. The trend is the same as the one observed in the experimentally obtained overall crystallization rate (1/τ_50%_), which indicates that Avrami theory is suitable to describe the crystallization of this polymer series, at least up to 50% conversion to the semicrystalline state.^18,19,32^

## 4 Conclusions

Comb polymers with alkane dithiol and propargyl PEG side chains have been investigated. The space between neighboring PEG side chains has been varied with the length of the spacers in the ring, and PEG chains of several molar masses have been employed. Comb-like polymers are able to crystallize under nonisothermal conditions, although the dithiol–yne-based ring backbone hinders the crystallization, which results in an increase in the amorphous layer thickness and thinner lamellae. The molar mass of the PEG side chains is the dominant factor in the melting and crystallization temperature. The crystallization and melting enthalpies are impacted by both molar mass of the PEG side chains and the tethering of the chain ends to the ring backbone.

The study of the crystallization kinetics indicates that the spherulitic and overall growth rates are reduced for comb-like polymers. This effect is more significant for low molar mass samples, which adopt an extended chain conformation. The tethering of one chain end to the ring backbone increases the overall crystallization energy barrier, confirming its hindering role in crystallization.

Regarding the microphase behavior obtained after isothermal crystallization of the cyclic comb-like polymers, it was verified that the tethering of PEG side-chains into a polythioether cyclic backbone, with distinct chain length between PEG side chains, drives a crystallization-induced self-assembly behavior into well-defined, highly ordered, semicrystalline superstructures. Evidence suggests that this is a consequence of interchain spatial constraints found between the PEG side chains, as several PEG chains must necessarily share the same interlamellar amorphous region due to the covalent connection with an essentially amorphous polythioether backbone. Besides, it was verified that the steric hindrance provided by PEG side-chains, with an extended backbone length separation within adjacent PEG of around 2.2 nm, leads to a majority of extended chain crystals for the isothermally crystallized cyclic comb-like polymer of PEG 5.1 kg/mol, although the analogous homopolymer presents solely chain-folded crystals. Increasing the length of the spacer in between PEG side chains for comb-like polymer of PEG 5.1 kg/mol releases the steric hindrance and allows the formation of a majority of interdigitated lamellar FCCs, which has not been reported before for comb polymers with PEG side chains.

Overall, this work demonstrates the impact of the backbone chain topology, the distance between neighboring side chains, and the length of the tethered side chains on the structure, lamellar morphology, and crystallization of comb-like polymers, a class of polymers that have been receiving increased interest from both academy and industry over the past decade.

## Supporting Information Available

Synthesis procedure and the molar mass information obtained by GPC; WAXS, SAXS, and DSC results; data corresponding to crystallinity degree obtained from DSC and WAXS and the calculated *l*_c_ and *l*_a_; PLOM micrographs taken during isothermal crystallization; data regarding crystallinity degree obtained by WAXS, long period, lamellar crystalline thickness, and amorphous layer; TEM micrographs of PEG 2.2 kg/mol crystallized isothermally; possible chain packing; TEM images of PEG 5.5 kg/mol crystallized isothermally; Avrami fitting example; and data of Avrami fittings (PDF)
