# Supplementary material for: Crystallization-Induced Self-Assembly of Poly(ethylene glycol) Side Chains in Dithiol–yne-Based Comb Polymers: Side Chain Spacing and Molecular Weight Effects
Source: Macromolecules. 2024 May 15;57(10):4906–17. doi: 10.1021/acs.macromol.4c00527 (PMC11140754; doi:10.1021/acs.macromol.4c00527)
Supplement: Supplementary file 1 — ma4c00527_si_001.pdf [file ma4c00527_si_001.pdf]

Supporting Information

Crystallization induced self-assembly of poly(ethylene glycol) side chains in dithiol-yne-based comb polymers: side chain spacing and molecular weight effects

Eider Matxinandiarena[1], Mario Iván Peñas[1], Brennan J. Curole[2], Monika Król[3], Lucas Polo Fonseca[1], Janne Ruokolainen[3], Scott M. Grayson[2], Leire Sangroniz[1], Alejandro J. Müller[1,4*]

[1]POLYMAT and Department of Polymers and Advanced Materials: Physics, Chemistry and Technology, Faculty of Chemistry, University of the Basque Country UPV/EHU, Paseo Manuel de Lardizábal, 3, 20018 Donostia-San Sebastián, Spain.

[2]Department of Chemistry, Tulane University, 6400 Freret Street, 2015 Percival Stern Hall, New Orleans, Louisiana 70118, United States

[3]Department of Applied Physics, School of Science, Aalto University, Espoo FIN-00076, Finland

[4]IKERBASQUE, Basque Foundation for Science, Plaza Euskadi 5, 48009, Bilbao, Spain.

Corresponding author: alejandrojesus.muller@ehu.es

Brief description of synthesis

The propargyl PEG and alkane dithiol were placed in a round-bottom flask and solubilized in methanol. A rubber septum was added, and the reaction flask was purged with argon gas. A stock solution of 2,2-dimethoxy-2-phenylacetophenone (DMPA) was made in methanol and was injected into the reaction flask. The solution was irradiated using a USpicy MACARON USND-3601 Professional UV Gel Lamp Nail Dryer (4 × 9 W 365 nm UV bulbs) for 30 min. The solvent was removed under reduced pressure, and the product was dissolved in DI water and washed with diethyl ether to remove unreacted thiol and with EtOAc to remove the DMPA. The comb polymer was extracted from the water via a separatory funnel using chloroform and a sodium chloride brine solution, dried with magnesium sulfate, and filtered. The solvent was removed under reduced pressure [1].

Table S1. Materials investigated and their corresponding data.

Comb Polymer	M_n by GPC (kDa)	M_w by GPC (kDa)	Đ by GPC
0.8-CP-BDT	11.5	27.1	2.36
2.2-CP-BDT	17.6	28.0	1.60
5.1-CP-BDT	31.9	36.5	1.14
0.8-CP-DDT	9.7	20.9	2.16
2.2-CP-DDT	23.4	35.9	1.53
5.1-CP-DDT	34.4	39.1	1.14

Non-isothermal crystallization

Wide-angle X-Ray Scattering (WAXS)

The crystalline structure of the materials was studied employing WAXS, see Figure S1. All the investigated systems show a semi-crystalline behavior, with well-defined peaks corresponding to the planes of the PEG crystal unit cell, indicating that even comb polymers can crystalize under non-isothermal conditions. PEG homopolymers display 2 main signals at 13.5 nm^{-1} and 16.5 nm^{-1}; the one around 13.6 nm^{-1} corresponds to the (111) and the 13.8 nm^{-1} to (120) planes. For the low molar mass 0.8 kg/mol sample, only the (120) plane is observed around 13.7 nm^{-1}, as the (111) plane reflection is absent. The signal around 16.5 nm^{-1} results from the combined scattering of the (032), (112), (132) and (212) planes [2, 3]. WAXS observations in Figure S1 revealed no shift of the diffraction peaks within the PEG homopolymers and comb-like polymers, regardless of the chain topology or the molecular weight of the PEG chains. This indicates that the crystalline unit cell of PEG chains does not vary in the comb polymers, and thus, the comb backbone is rejected to the amorphous region.

PEG crystals have a monoclinic unit cell with a = 0.805 nm, b = 1.304 nm, and c = 1.948 nm, and β = 125.4º [4-6]. The chains adopt a 7_2 helical conformation [4, 5]. In the case of PEG 0.8 kg/mol, the homopolymer showed very low-intensity peaks, which was attributed to experimental issues due to the physical nature of the sample, which was liquid at room temperature. The degree of crystallinity has been determined from WAXS diffractograms, and the results are depicted in Table S2 in the Supporting Information. The crystallinity degree increases with molar mass reaching a saturation level above 2.2 kg/mol, as could be expected [7]. The comb polymers show a reduced crystallinity degree, which may indicate that the tethering of one chain end to the ring backbone hinders the crystallization process.

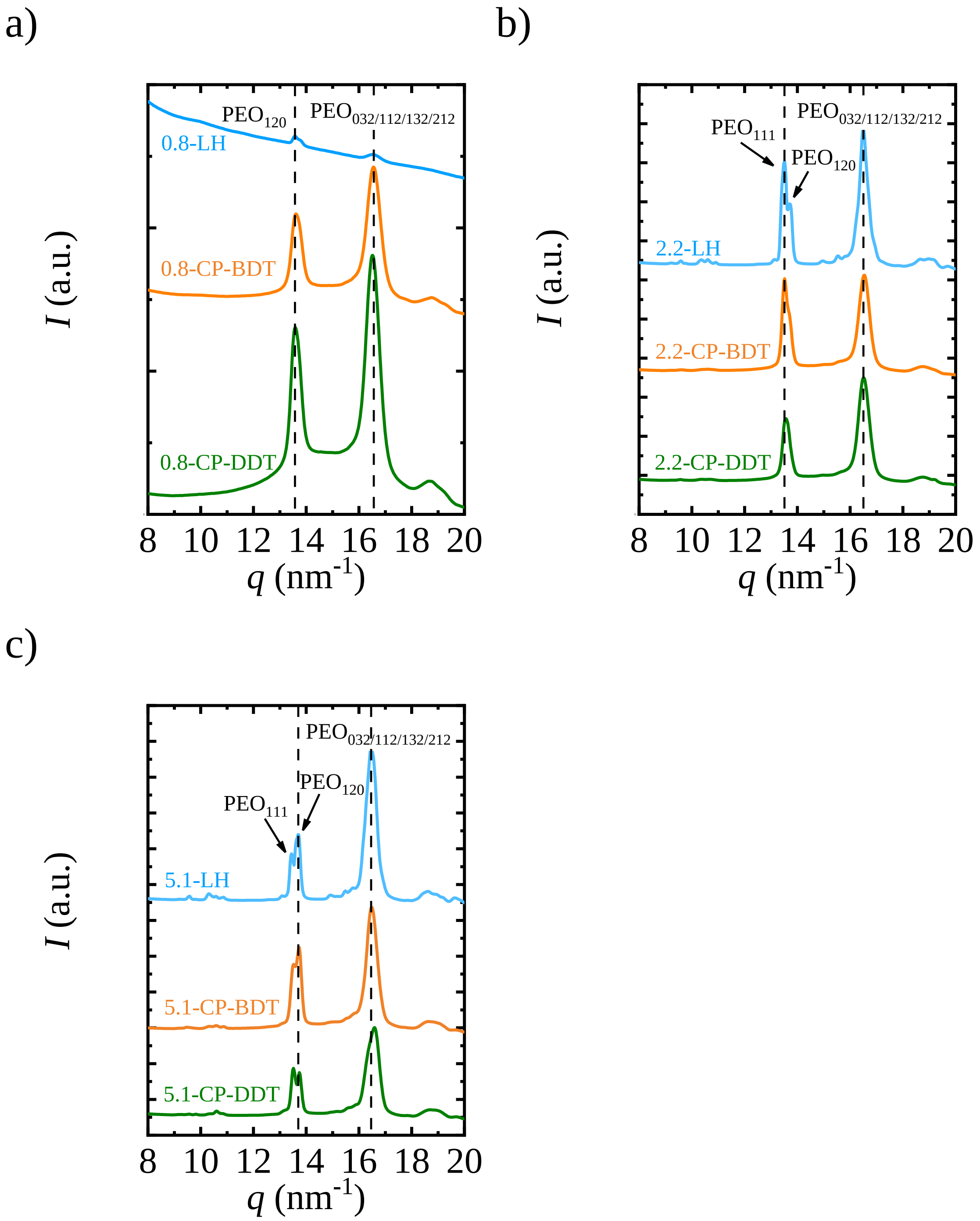

Figure S1. WAXS patterns at -40 °C of the materials investigated: a) 0.8 kg/mol, b) 2.2 kg/mol, and c) 5.1 kg/mol.

Small-angle X-Ray Scattering (SAXS)

After crystallizing the samples, by cooling them from the melt at 20 °C/min down to -40 °C, SAXS experiments were performed. Figure S2 shows the SAXS curves obtained at -40 °C for the studied polymer families.

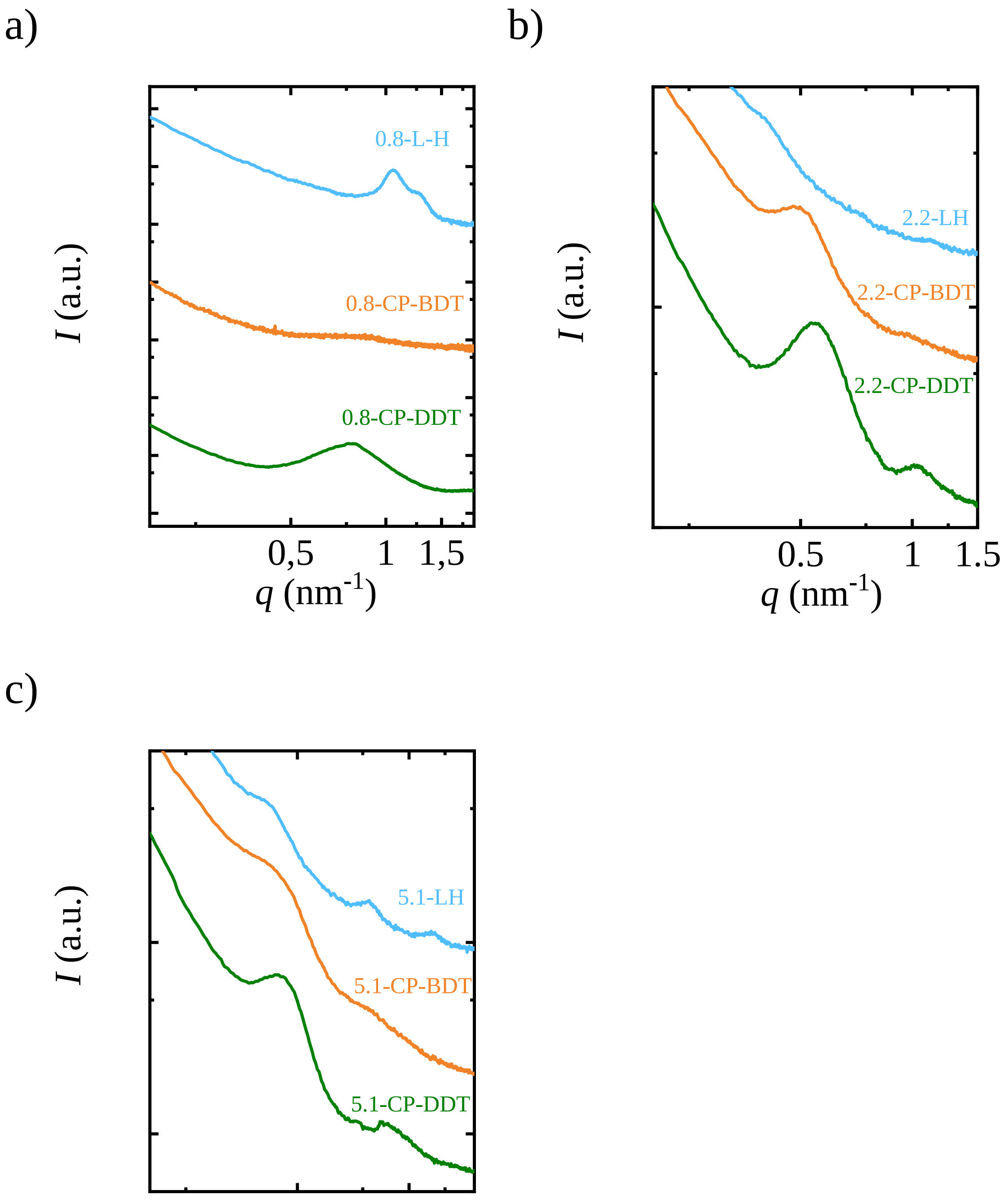

Figure S2. SAXS curves obtained by crystallizing the sample upon cooling from the melt at 20 °C/min. Data corresponding to the linear homopolymer, comb polymer containing BDT units and DDT units in the ring are shown for a) 0.8 kg/mol PEG chain length, b) 2.2 kg/mol, and c) 5.1 kg/mol.

The linear PEG polymers with 2.2 and 5.1 kg/mol have three characteristic peaks that correspond to the first, second, and third order reflections (from the lowest to the

highest *q* value), indicating very good lamellar stacking. Only the two primary reflections are observed when PEG chains are linked to the ring backbone (forming a comb polymer), indicating slightly less order in lamellar packing. The linear 0.8 kg/mol PEG homopolymer and the corresponding comb polymers show one maximum, which reflects a system with a more limited order than higher molar mass samples.

Considering the position of the maxima (*q* value), Bragg´s law can be used to determine the long period, which is comprised of the amorphous layer and the crystalline lamella. The long period is inversely proportional to the *q* value; thus, the shift observed to higher *q* values when PEG chains are covalently linked to the ring backbone indicates a reduction in the long period, which will be explained below.

Depending on the molar mass, PEG chains can adopt an extended or chain-folded conformation in the lamellar crystals. PEG with low molar mass forms extended chain crystals (ECC) [7, 4, 8, 9]. Increasing the molar mass, i.e., the length of the PEG chains, results in a transition to folded chain crystals (FCC). The molar mass at which this transition occurs depends on the conditions employed to crystallize the samples. To determine if the PEG side chains form ECC or FCC lamellar crystals, it should be noted that the PEG crystallizes in monoclinic unit cells, as proved by WAXS, and that in this form, PEG chains adopt a *7/2* helix conformation with a *c*-axis length of 1.984 nm [4, 5, 10, 11].

Taking into account the number of monomer units in each chain, i.e., the molar mass, the length that the chains would have in an extended conformation in the lamellar crystal can be calculated (considering its minimum energy conformation inside the crystalline structure with *7/2* helix packing), and this value can be compared with the experimentally measured long period in Figure 4 in the main manuscript [11].

Figure 4 in the main manuscript shows that the 2.2-LH linear PEG homopolymer has a long period value very close to the estimated length of an extended chain, which indicates that these PEG chains probably form ECC lamellar crystals for this low molar mass sample. When the degree of crystallinity is high (which is usually the case for low molar mass PEG chains), the extended chain length (inside the lamellar crystal) can be directly compared with the long period as the amorphous intervening layer (l_a) in between the crystalline lamellae is very small.

On the contrary, 5.1-LH PEG has a much lower long period value than the one corresponding to the extended chain length inside the crystal (see Figure 4), confirming that the chains fold and form FCC lamellar crystals for this molar mass. A slight reduction in the long period is observed for PEG comb polymer samples with side chains of 2.2 and 5.1 kg/mol in Figure 4. For comb polymers with 2.2 kg/mol chains could form ECC lamellar crystals with entire or partial chain interdigitation. In the case of comb polymers with 5.1 kg/mol PEG side chains, both folding and partial interdigitation are possible. The PEG linear homopolymer with 0.8 kg/mol shows a clear maximum at q=1.06 nm^{-1} in the SAXS measurements. Furthermore, the comb polymers with PEG 0.8 kg/mol side chains have a long period compatible with ECC lamellar crystals (see Figure 4). This indicates that the chains adopt extended chain conformations, as several chains form ECC lamellae; they probably adopt an interdigitated chain packing in the crystal. However, it is clear from Figure 4, that at this low molar mass, 0.8 kg/mol, the PEG chains either in the homopolymer or in the combs do not form FCC lamellar crystals.

Considering the crystallinity degree of the samples and the long period, it is possible to estimate the crystalline lamellar thickness (l_c) and the thickness of the amorphous layer (l_a), considering $l_c = d \cdot X_v$ and $d = l_a + l_c$. Figure S3 shows the l_c and l_a values for the linear and comb-like polymers. The linear polymers show thicker crystalline lamellae and thinner amorphous layers resulting from the high crystallinity degree. The lamellar thickness, l_c, slightly decreases when the chain length increases from 2.2 to 5.1 kg/mol PEG homopolymers (see Figure S3), as most of the lamellar crystals in the 2.2 kg/mol case are ECC, while for the 5.1 kg/mol they are FCC lamellae.

When PEG chains are covalently linked to the dithiol-yne-based ring backbone, the crystalline lamellar thicknesses are reduced significantly, whereas the amorphous layer becomes thicker. Increasing the length of alkanedithiol spacers (distance between PEG side chains) from 4 to 10 methylene groups does not cause a significant effect on the values of l_c and l_a, which indicates that the molar mass and chain topology (linear versus comb) seem to be the dominant factors in determining the PEG lamellar morphology.

These results are in agreement with similar reported studies for other types of comb PEGs containing a poly(norbornene) (PNB) backbone with PEG side chains of 3 and 6 kg/mol, which have shown that tethering one PEG chain end to a norbornene unit

or to the poly(norbornene) backbone reduces slightly the crystalline lamellar thickness (around 10 %) and increases the amorphous layer which arises from the restrictions imposed to the PEG chains [12].

The factors governing lamellar thickness values have been studied in depth by Thurn-Albrecht et al. [13-17]. They have shown that lamellar thickness depends on crystal growth and intracrystalline chain diffusion, i.e., the movement of conformational defects along the crystal, which leads to crystal thickening. PEG is a crystal-mobile polymer, i.e., reorganization occurs in the same time scale as crystal growth under isothermal conditions. The studies carried out by NMR and SAXS techniques indicate that the thickness of the crystalline lamellae increases until the amorphous region reaches a specific minimum value, which results in well-defined amorphous layer thickness [13-16]. More recent studies with PCL, which is a crystal-fixed polymer (well-defined crystalline lamellae), have revealed the role of entanglements on the amorphous layer thickness [17].

In comb polymers, one of the PEG chain ends is tethered to the backbone, which reduces the flexibility and diffusion of the chain. Additionally, neighboring PEG atoms to the point where the chains are tethered to the cyclic part of the polymer will be rejected to the amorphous intercrystalline lamellar regions. Rejecting the entire cycle with part of the tethered segments to the amorphous regions becomes a driving force for self-assembly as the material crystallizes. Chain tethering will thus produce thinner lamellae during non-isothermal crystallization because of reductions in crystallinity compared to neat PEG chains due to mobility restrictions and the larger amount of material rejected to the amorphous part of the spherulites.

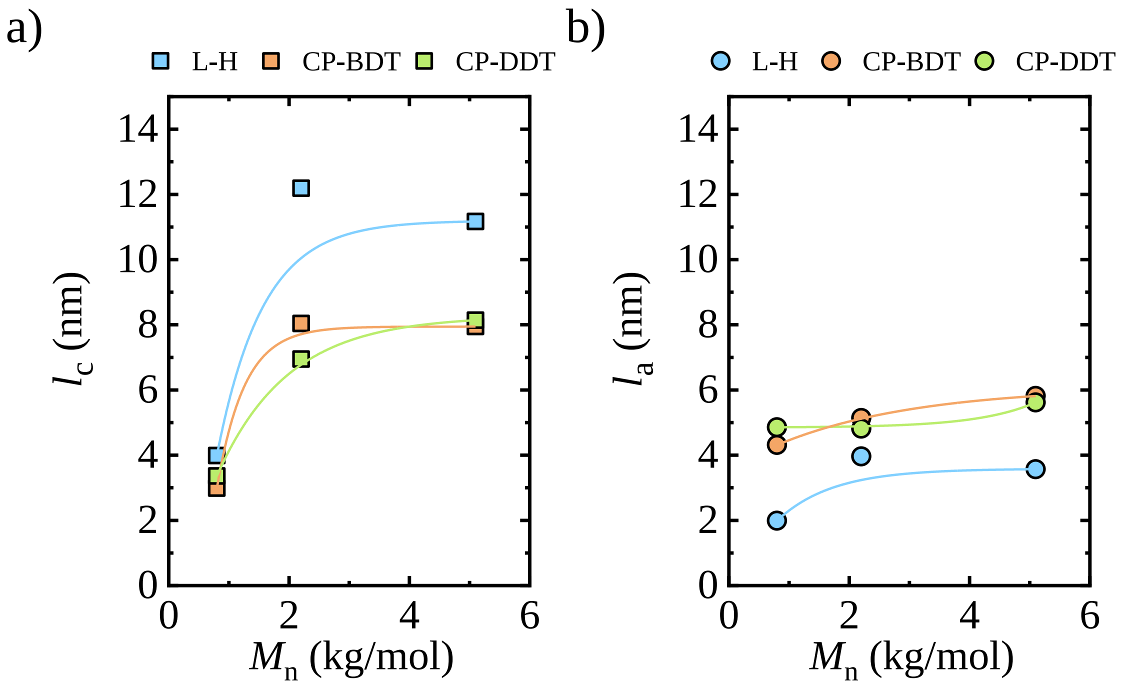

Figure S3. a) Crystalline lamellar thickness and b) amorphous layer thickness for the samples investigated in this work. Crystallinity degree values from WAXS measurements were employed to calculate l_c. The solid lines are arbitrary guides to the eyes.

Non-isothermal crystallization by DSC

DSC experiments were performed to study the samples' non-isothermal crystallization and melting behavior. Figure S4 shows the cooling scans from the melt and subsequent heating scans of PEG linear homopolymers and comb polymers containing different spacers in the ring backbone.

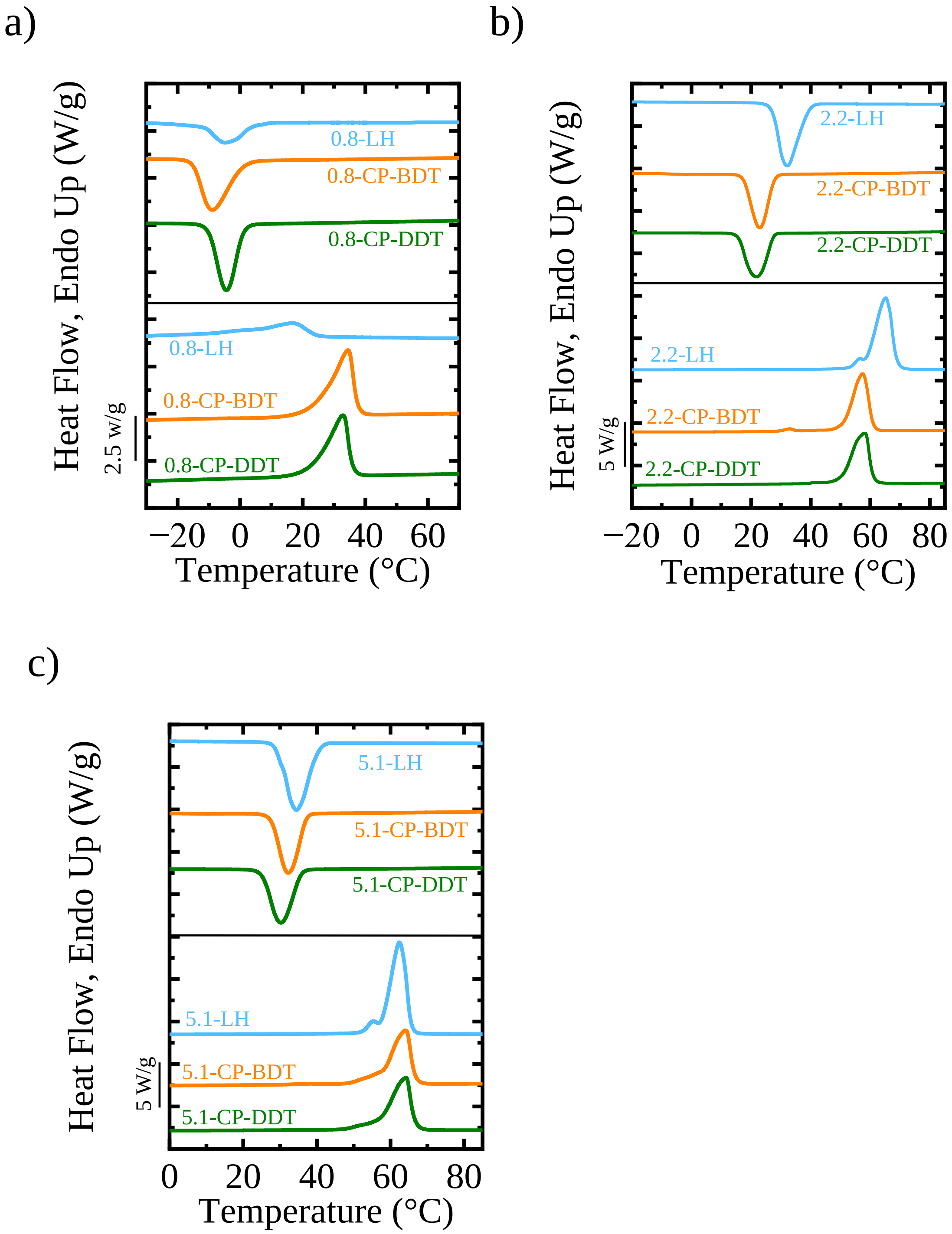

Figure S4. DSC cooling (upper side of the graphs) and subsequent heating scans at 20 °C/min (lower side of the graphs) of LH, CP-BDT and CP-DDT samples for: a) 0.8 kg/mol, b) 2.2 kg/mol, and c) 5.1 kg/mol.

All the investigated samples show an exothermic peak in the cooling scans, indicating that the samples can crystallize during cooling at 20 °C/min. The subsequent heating scans show the corresponding melting endotherms. Interestingly, PEG

homopolymers in Figure S4, show some peculiarities, as the 2.2 kg/mol and 5.1 kg/mol samples display a small melting peak that overlaps with the main endothermic event and a very broad melting endotherm for the 0.8 kg/mol PEG. The double melting may be related to the co-existence of FCC and ECC lamellar crystals.

Even though Figure S3 shows that the average lamellar thickness indicates that the 0.8 and 2.2 kg/mol samples crystallize with ECC lamellae and the 5.1 kg/mol sample with FCC lamellae, a mixture of ECC with a small amount of FCC could also be formed for the first two samples and vice versa for the last one, especially during cooling from the melt at 20 ºC/min. A reorganization process during the scan can also explain the appearance of this bimodal melting behavior. Polymorphism can be ruled out according to our WAXS experiments presented above.

Figure S5 presents the crystallization and melting temperatures as a function of the molar mass to understand the effect of having PEG chains linked covalently to a dithiol-yne-based ring backbone. An increase in T_c is observed with the molar mass until a constant value is reached above 2 kg/mol [7]. The comparison of linear and comb-like polymers revealed that the T_c is reduced very slightly for the comb-like polymers, except for the PEG 2.2 kg/mol sample. For this sample, a significant reduction in the T_c is observed when one chain end is linked to the ring backbone. Considering that the equilibrium melting temperature is kept constant (irrespective of whether the PEG chains are attached to a backbone or not), the results may reflect that higher undercoolings are required for crystallization when the PEG arms are attached to a ring backbone in comparison with free chain ends of the PEG linear polymer.

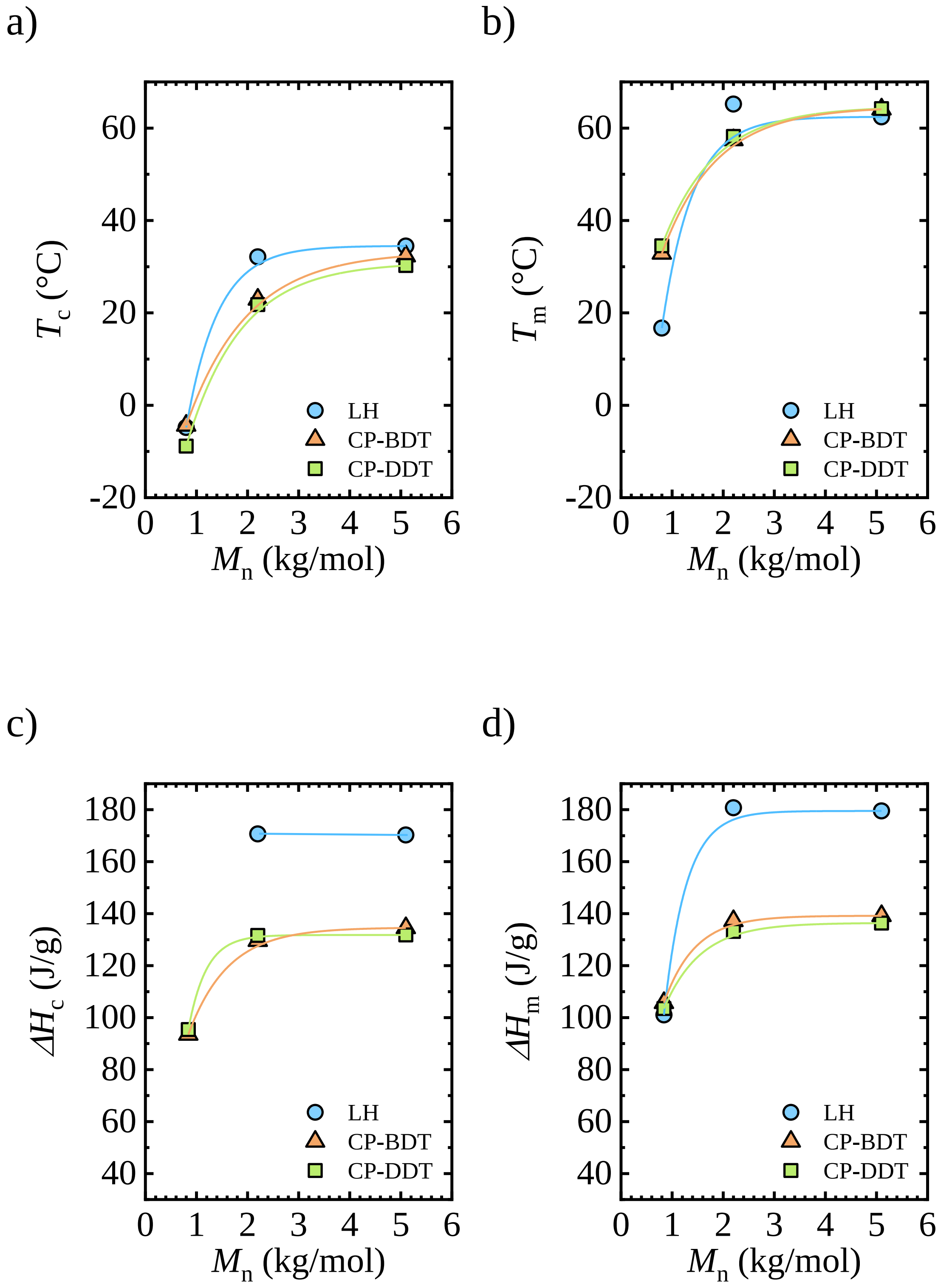

Figure S5. a) Peak crystallization temperature, b) peak melting temperature, c) normalized crystallization enthalpy, and d) normalized melting enthalpy as a function of molar mass for the linear, and comb polymers with the monopropargyl PEG with BDT and DDT spacers. The solid lines are arbitrary guides to the eyes.

A similar trend is observed with T_m, as linear homopolymers and comb-like polymers reach a saturation level above 2 kg/mol, as could be expected from the general trend of increasing melting point with molar mass [7]. The low T_m value of 0.8 kg/mol results from the depression provoked by the chain ends that act as defects [18-21]. This effect is not observed for higher molar masses PEG samples since, when the chain length is increased, the concentration of chain ends is reduced, so their effect becomes negligible. For extended chain crystals, the T_m also depends on the lamellar thickness which is proportional to the chain length [21-23].

Analyzing the effect of tethering PEG chains to a ring backbone, different trends are observed depending on the molar mass of PEG chains. The shortest PEG chains, 0.8 kg/mol, show an increase in T_m when the PEG chains are linked to the ring backbone. PEG 2.2 kg/mol shows a significant T_m reduction when chain ends are linked to the ring backbone whereas the reduction is very subtle for PEG 5.1 kg/mol. SAXS results indicate that there is a reduction of l_c for comb polymers with 2.2 kg/mol and 5.1 kg/mol PEG side chains. This reduction of l_c is more significant for PEG 2.2 kg/mol, which could explain the higher depression observed in T_m for this sample in which chains mostly form ECC lamellar crystals. On the contrary, in PEG 5.1 kg/mol, the chains adopt a folded conformation (FCC), so the lamellar thickness has a weaker effect in the T_m.

The crystallization and melting enthalpy show a monotonic increase with molar mass, as could be expected. For 0.8 kg/mol there is an increase in the melting enthalpy when PEG chains are tethered to the cyclic backbone. The crystallization enthalpy of 0.8-LH obtained by integration of the crystallization exotherm, gave unreliable values, as the crystallization peak was too small and very broad, therefore it is not reported. On the contrary, higher molar mass PEG samples show a reduction in both enthalpies for comb-like polymers, indicating a restriction imposed by the covalent tethering of one chain end, which hinders the crystallization process and lower crystallinity degrees result.

Studies carried out with PEG of different architectures comparing linear, star, and brushes with molar mass ranges between 1 and 4 kg/mol have reported an increase in T_m with branching degree, whereas the T_c and crystallinity were reduced [24]. The authors did not correlate the results with the lamellar thickness because SAXS experiments were not performed in the above-cited references [24-25].

Comparison of long period, crystalline lamellae and amorphous layer obtained under non-isothermal crystallization

Comparison of X_v values from DSC or WAXS to consider the error from the measurement technique in the crystalline lamellar thickness and amorphous layer thickness.

Table S2. Crystallinity degrees obtained from DSC and WAXS in weight (X_c) and in volume (X_V), long period form SAXS (d) and calculated crystalline lamellar thickness (l_c) and amorphous layer (l_a).

Sample	$X_{c, DSC}$ (%)	$X_{c,WAXS}$ (%)	$X_{V, DSC}$ (%)	$X_{V,WAXS}$ (%)	d_{SAXS} (nm)	$l_{c, DSC}$ (nm)	$l_{a, DSC}$ (nm)	$l_{c, WAXS}$ (nm)	$l_{a, WAXS}$ (nm)
0.8-LH	47.2	68.9	44.7	66.7	5.98	2.67	3.31	4.0	2.0
2.2-LH	84.4	77.3	83.0	75.5	16.2	13.4	2.7	12.2	4.0
5.1-LH	83.9	77.6	82.5	75.8	14.7	12.2	2.6	11.2	3.6
0.8-CP-BDT	43.1	43.4	40.6	40.9	7.3	3.0	4.3	3.0	4.3
2.2-CP-BDT	60.7	63.4	58.2	61.0	13.2	7.7	5.5	8.0	5.1
5.1-CP-BDT	63.5	60.2	61.1	57.7	13.8	8.4	5.4	7.9	5.8
0.8-CP-DDT	38.8	43.4	36.4	40.9	8.2	3.0	5.2	3.4	4.9
2.2-CP-DDT	56.9	61.5	54.4	59.1	11.8	6.4	5.4	6.9	4.8
5.1-CP-DDT	61.2	61.6	58.8	59.2	13.8	8.1	5.7	8.1	5.6

Isothermal crystallization

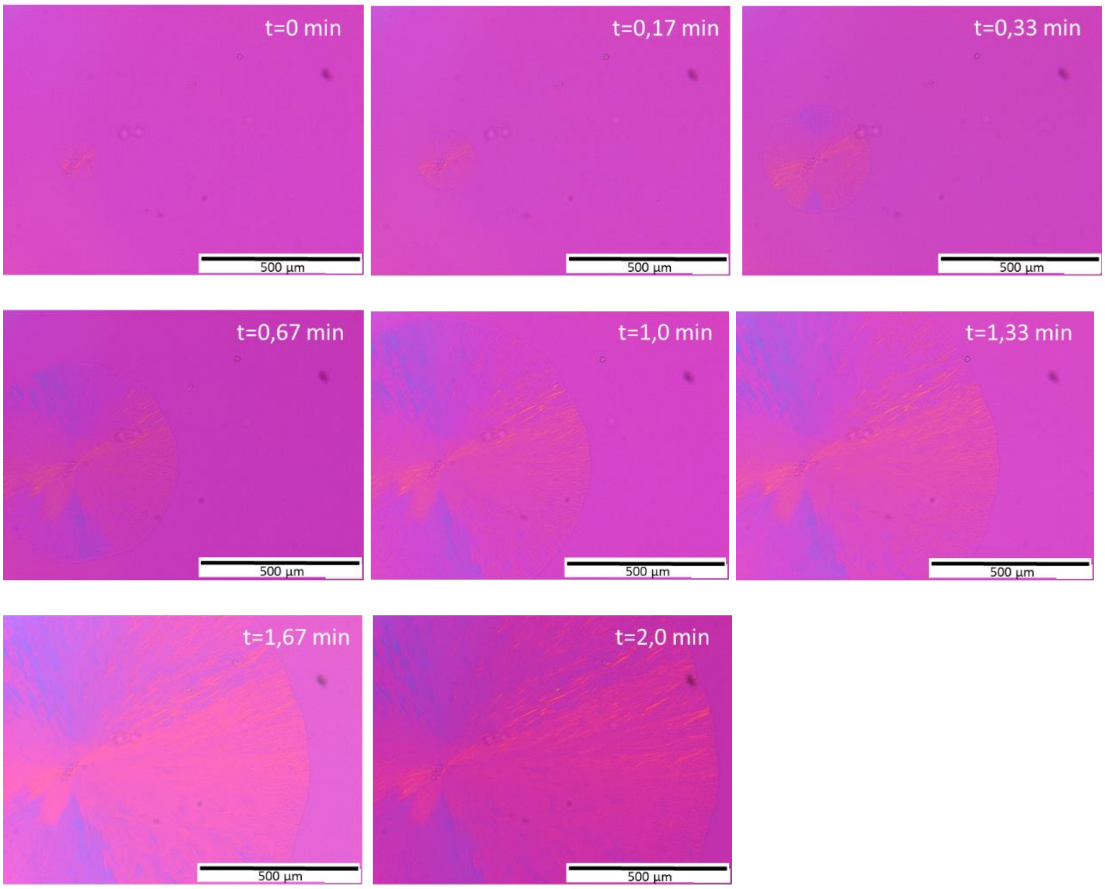

Figure S6. PLOM micrographs of 2.2-LH at 48 °C acquired at several times.

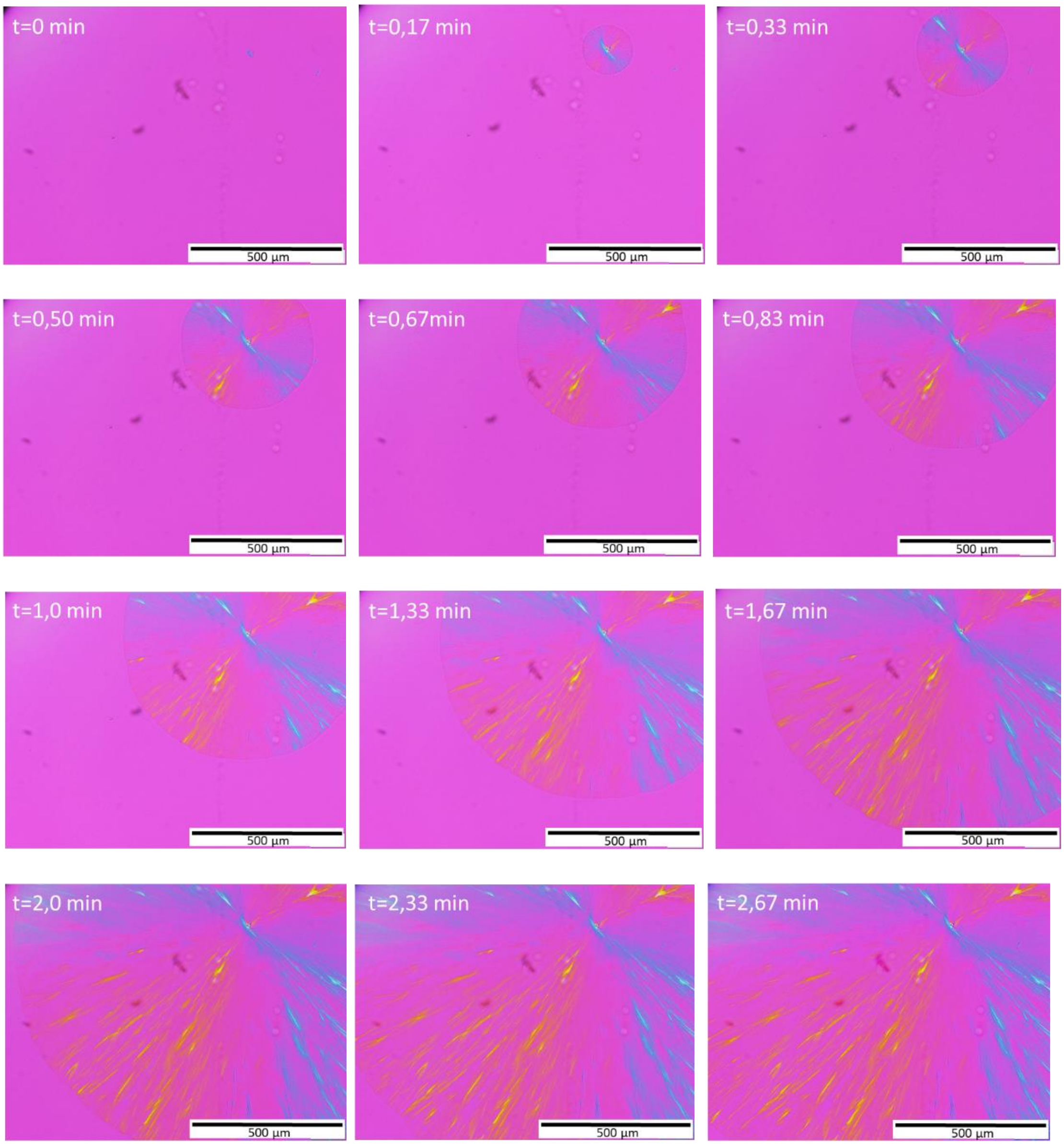

Figure S7. PLOM micrographs of 5.1-LH at 48 °C acquired at several times.

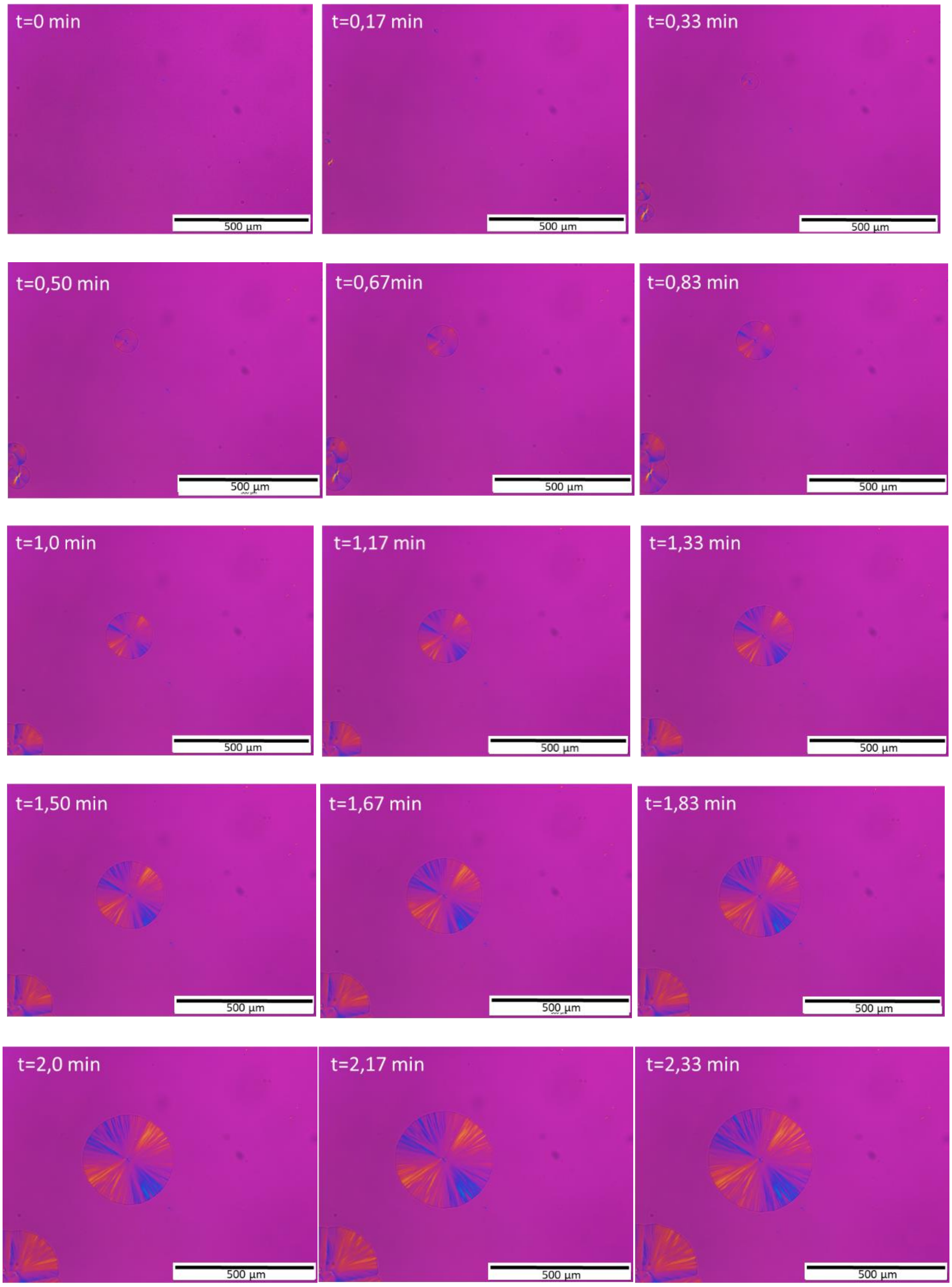

Figure S8. PLOM micrographs of 2.2-CP-BDT at 37 °C acquired at several times.

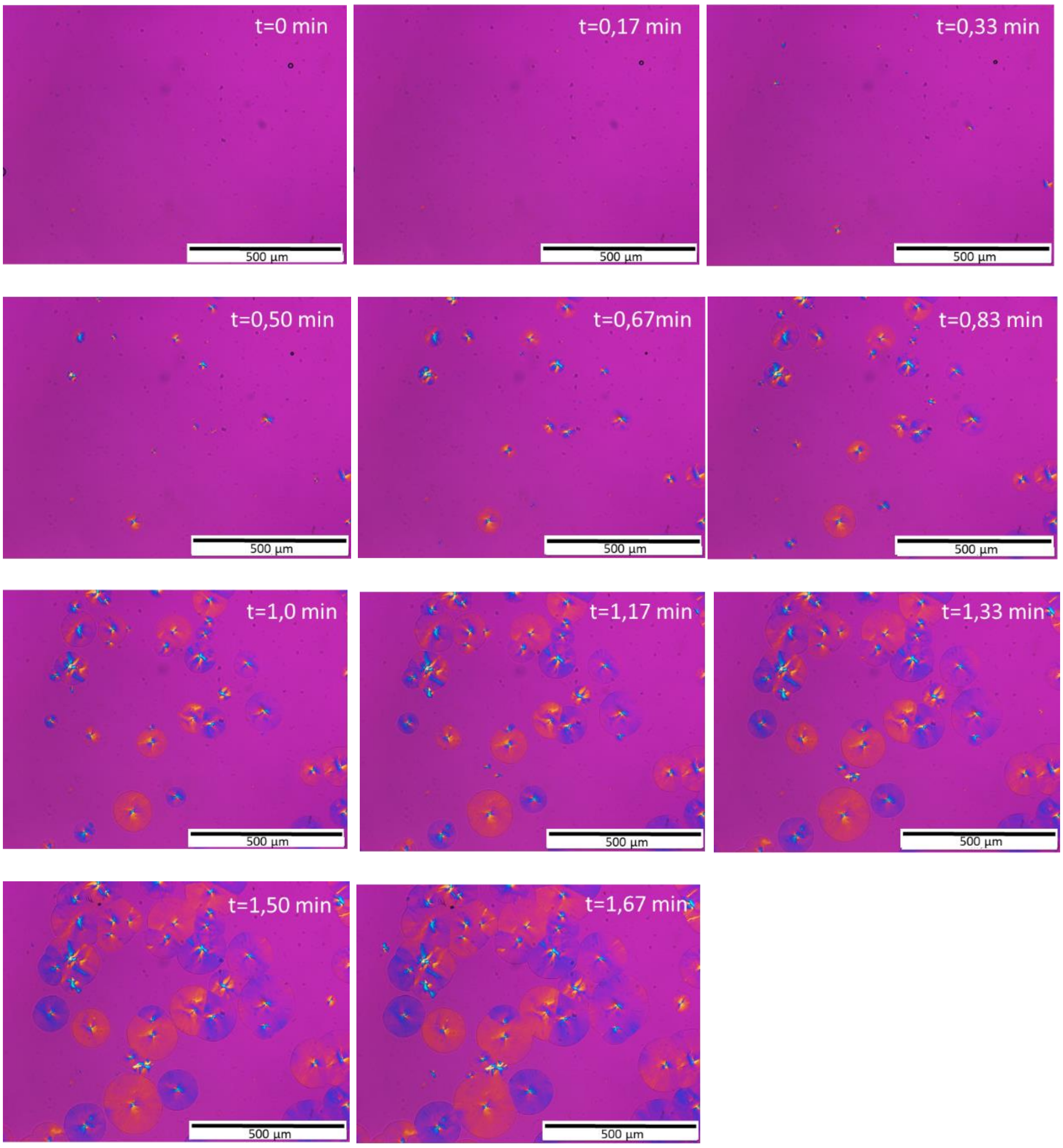

Figure S9. PLOM micrographs of 5.1-CP-BDT at 47 °C acquired at several times.

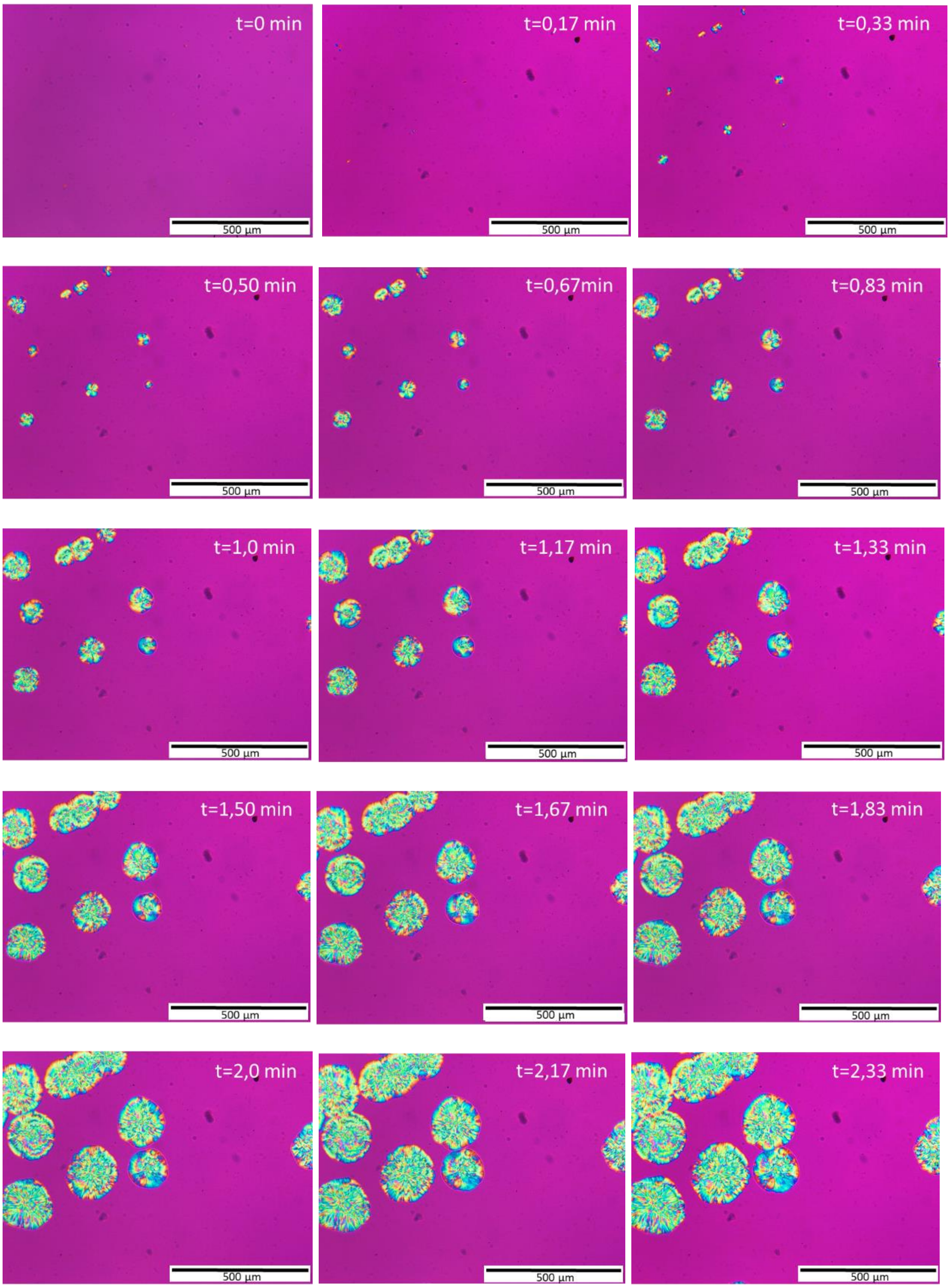

Figure S10. PLOM micrographs of 2.2-CP-DDT at 37 °C acquired at several times.

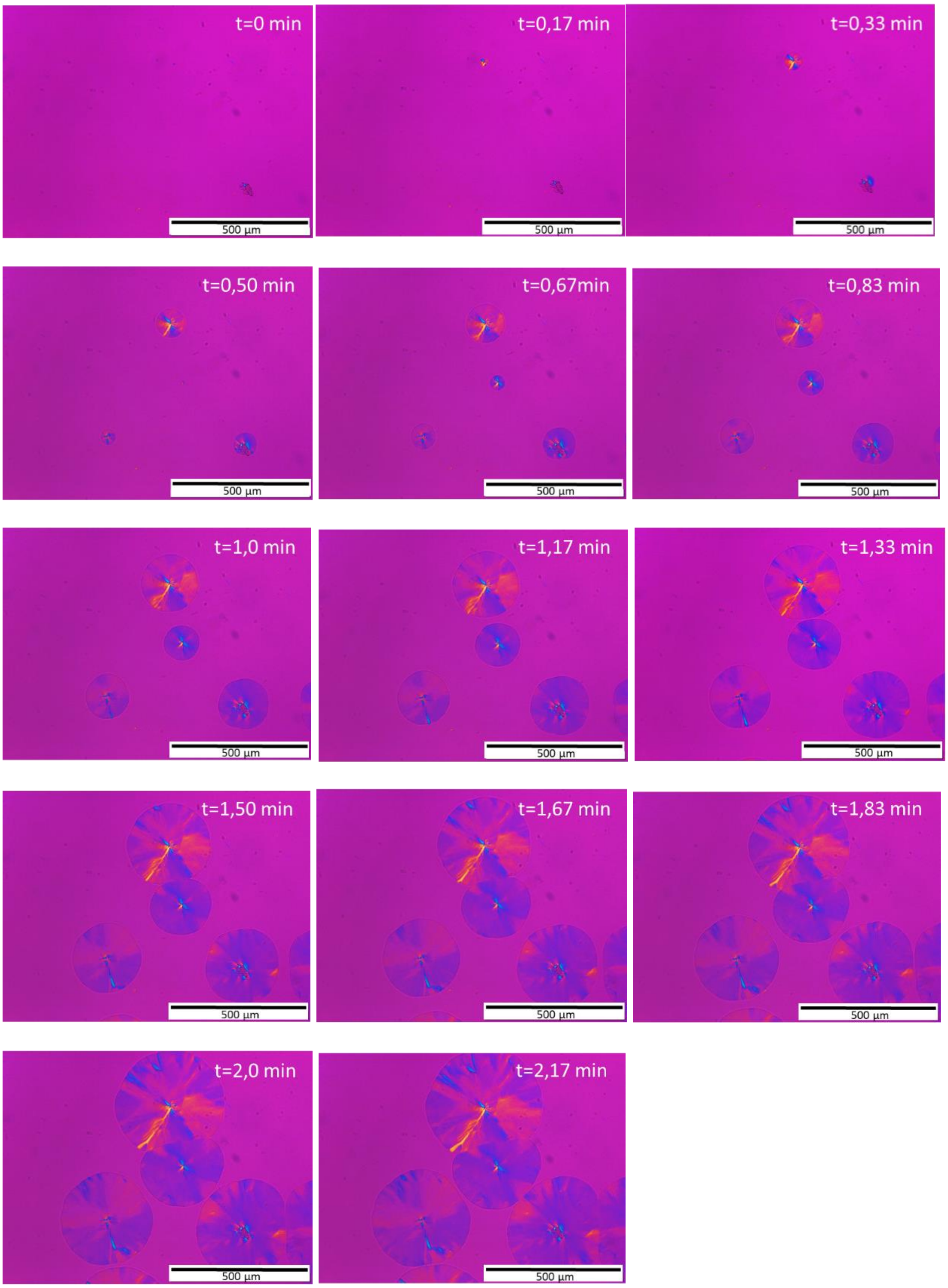

Figure S11. PLOM micrographs of 5.1-CP-DDT at 45 °C acquired at several times.

Table S3. Crystallinity degrees obtained from WAXS by weight (X_c) and by volume (X_V), long period from SAXS (d) and calculated crystalline lamellar thickness (l_c) and amorphous layer thickness (l_a).

Sample	$X_{c,WAXS}$ (%)	$X_{V,WAXS}$ (%)	d_{SAXS} (nm)	$l_{c,\ WAXS}$ (nm)	$l_{a,\ WAXS}$ (nm)
2.2-LH	78.2	76.4	16.01	12.2	3.8
2.2-CP-BDT	69.1	66.9	13.76	9.2	4.6
2.2-CP-DDT	63.8	61.4	13.76	8.5	5.3
5.1-LH	79.2	77.5	16.44	12.7	3.7
5.1-CP-BDT	60.3	57.8	23.51	13.6	9.9
5.1-CP-DDT	67.5	65.2	12.38	8.1	4.3

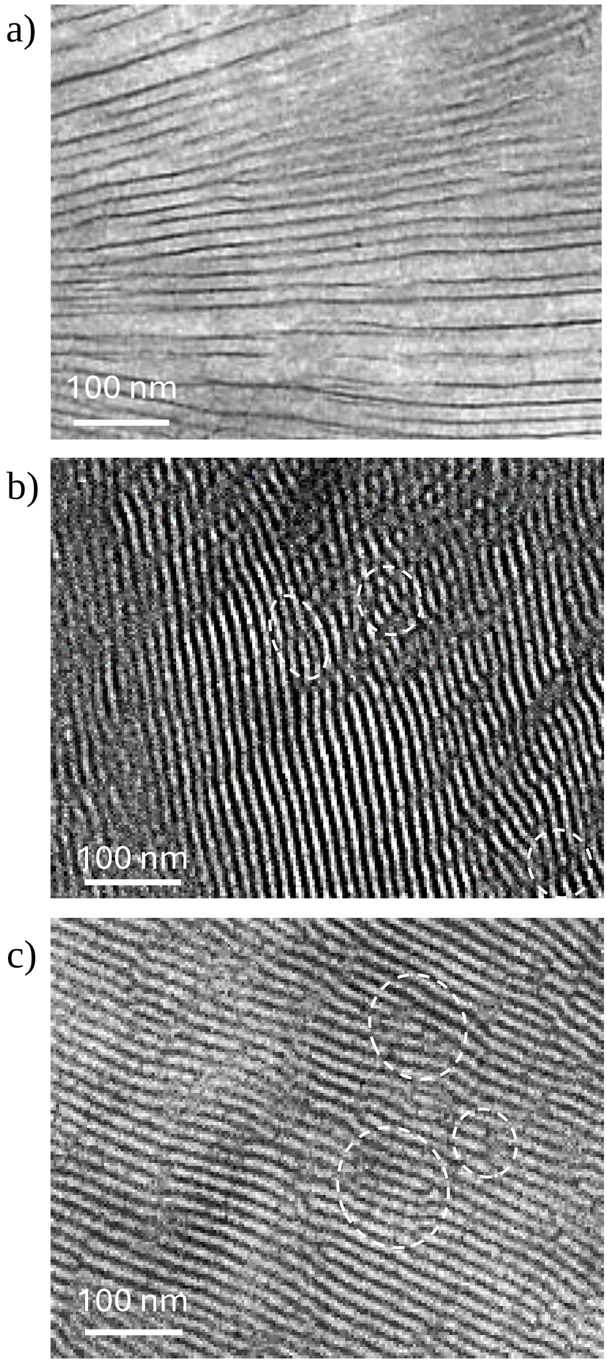

Figure S12. TEM images of a) 2.2-LH, b) 2.2-CP-BDT, and c) 2.2-CP-DDT, acquired with Ru-stained samples after isothermal crystallization.

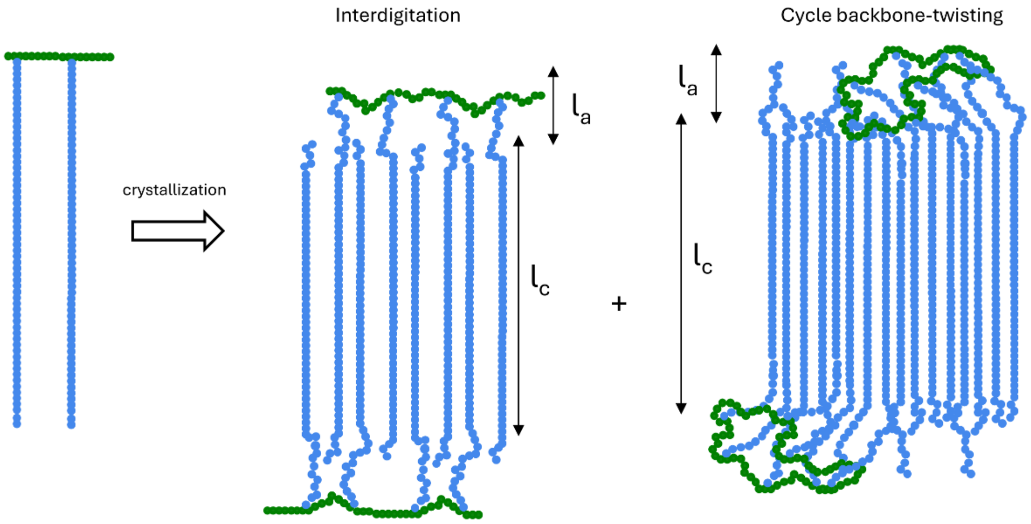

Figure S13. Model showing possible chain packing scenarios after crystallization: interdigitation and cycle backbone-twisting.

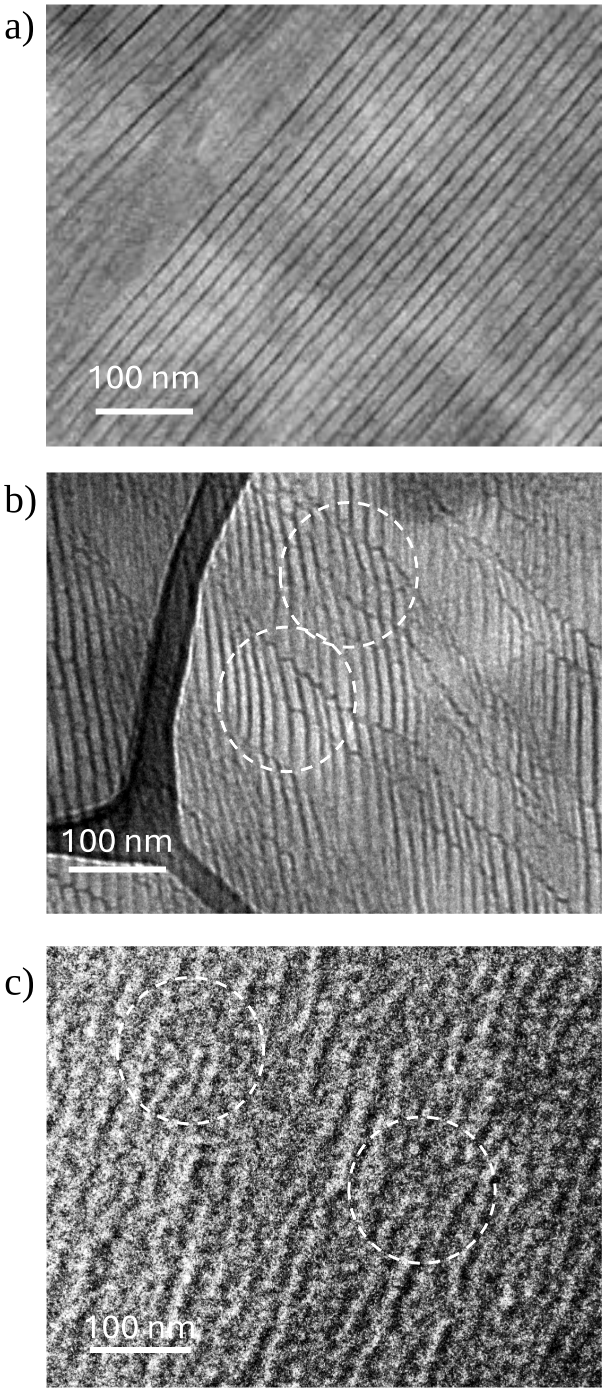

Figure S14. TEM images of a) 5.1-LH, b) 5.1-CP-BDT, and c) 5.1-CP-DDT, acquired with Ru-stained samples after isothermal crystallization.

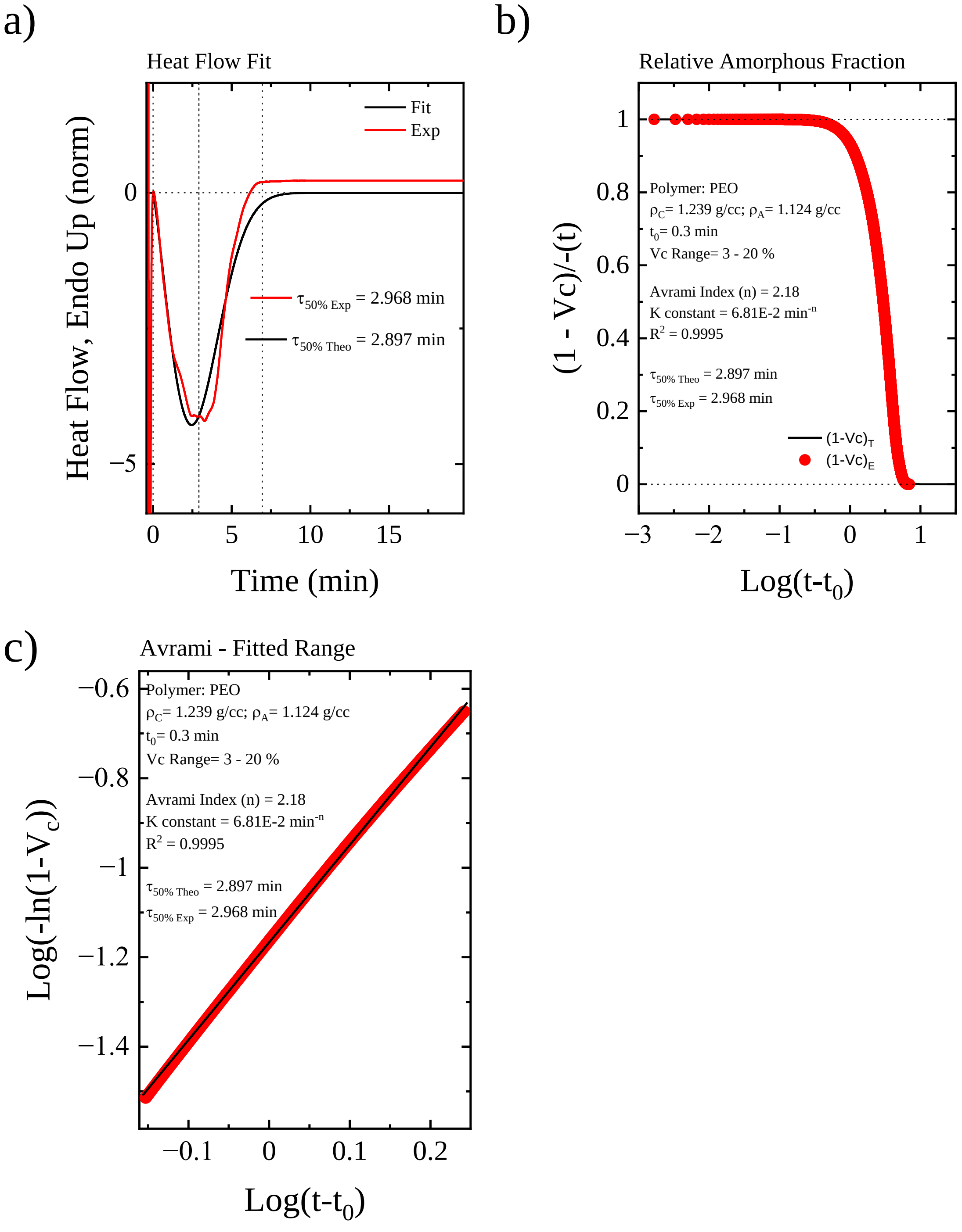

Figure S15. Avrami fitting of 5.1-CP-BDT sample at 43 °C. Experimental and fitted curve of a) the isothermal crystallization, b) relative amorphous fraction and c) linearized Avrami plot.

Table S4. Parameters obtained from Avrami theory fitting the isothermal experiments carried out in the DSC for linear homopolymers.

	T_c (°C)	Avrami Index (n)	$K^{(1/n)}$	R^2	$\tau_{50\%}$ (theo)	$\tau_{50\%}$ (exp)	$1/\tau_{50\%}$ (exp)
2.2-LH	44	2.19	0.322	0.9995	2.624	2.452	0.407
	44.5	2.28	0.274	0.9997	3.115	2.923	0.342
	45	2.21	0.221	0.9997	3.816	3.502	0.285
	45.5	2.33	0.178	0.9996	4.774	4.422	0.226
	46	2.59	0.149	0.9997	5.814	5.863	0.170
	46.5	2.22	0.109	0.9986	7.762	6.798	0.147
	47	2.23	0.083	0.9977	10.174	9.022	0.110
	47.5	2.13	0.098	0.9994	8.508	9.978	0.100
	48	2.17	0.134	0.9990	6.309	7.342	0.136
	48.5	2.51	0.133	0.9983	6.463	7.940	0.125
	49	2.54	0.065	0.9999	11.862	15.288	0.065
	49.5	2.36	0.053	0.9959	13.658	15.010	0.066
5.1-LH	46.5	2.03	0.253	0.9998	3.303	3.388	0.295
	47	1.96	0.202	0.9995	4.091	4.223	0.236
	47.5	2.04	0.177	0.9982	4.700	5.018	0.199
	48	2.07	0.113	0.9948	7.383	7.873	0.127
	48.5	1.81	0.085	0.9985	9.605	9.797	0.102
	49	2.19	0.069	0.9996	12.173	12.173	0.082
	49.5	1.97	0.044	0.9990	18.596	19.558	0.051

Table S5. Parameters obtained from Avrami theory fitting the isothermal experiments carried out in the DSC for comb polymers with BDT spacers (four methylene carbons).

	T_c (°C)	Avrami Index (n)	$K^{(1/n)}$	R^2	$\tau_{50\%}$ (theo)	$\tau_{50\%}$ (exp)	$1/\tau_{50\%}$ (exp)
0.8-CP-BDT	23	1.98	0.772	0.9993	0.947	1.097	0.911
	23.5	2.19	0.301	0.9980	1.462	1.775	0.563
	24	2.14	0.151	0.9987	2.040	2.393	0.418
	24.5	2.44	0.056	0.9983	2.790	3.252	0.307
	25	2.49	0.016	0.9978	4.513	5.322	0.188
	25.5	2.64	0.005	0.9980	6.622	7.668	0.130
2.2-CP-BDT	31.5	2.09	0.362	0.9987	2.316	2.760	0.362
	32	2.42	0.361	0.9985	2.376	2.818	0.354
	32.5	2.64	0.334	0.9989	2.601	3.438	0.290
	33	2.23	0.209	0.9917	4.043	4.925	0.203
	33.5	2.66	0.181	0.9992	4.791	5.370	0.186
	34	2.22	0.135	0.9903	6.280	7.128	0.140
	34.5	3.07	0.134	0.9948	6.594	8.212	0.121
	35	2.56	0.101	0.9921	8.554	10.008	0.099
	35.5	2.80	0.093	0.9905	9.366	11.880	0.084
	36	2.59	0.067	0.9873	12.895	16.027	0.062
	36.5	2.90	0.054	0.9917	16.161	20.375	0.049
5.1-CP-BDT	42	2.02	0.150	1.0000	1.850	1.798	0.556
	42.5	1.97	0.339	0.9998	2.440	2.370	0.421
	43	2.18	0.291	0.9995	2.897	2.968	0.336
	43.5	1.91	0.244	0.9977	3.378	3.500	0.285
	44	1.93	0.172	0.9992	4.773	4.708	0.212
	44.5	2.15	0.154	0.9989	5.481	5.880	0.170
	45	2.22	0.138	0.9989	6.169	6.813	0.146
	45.5	2.35	0.103	1.0000	8.277	8.162	0.122
	46	2.66	0.0889	0.9994	9.774	10.708	0.093
	46.5	2.67	0.071	1.0000	12.198	12.902	0.077
	47	2.94	0.057	0.9999	15.367	15.487	0.064

Table S6. Parameters obtained from Avrami theory fitting the isothermal experiments carried out in the DSC for comb polymers with DDT spacers (ten methylene carbons).

	T_c (°C)	Avrami Index (n)	$K^{(1/n)}$	R^2	$\tau_{50\%}$ *(theo)*	$\tau_{50\%}$ *(exp)*	$1/\tau_{50\%}$ *(exp)*
0.8-CP-DDT	21	2.13	0.903	0.9998	0.932	1.017	0.983
	21.5	2.15	0.712	0.9997	1.184	1.317	0.759
	22	2.54	0.536	0.9990	1.612	1.887	0.529
	22.5	2.83	0.426	0.9982	2.061	2.444	0.409
	23	2.51	0.323	0.9992	2.676	3.140	0.318
	23.5	3.49	0.254	0.9953	3.542	4.318	0.231
	24	2.97	0.201	0.9988	4.394	5.063	0.197
2.2-CP-DDT	30.5	2.11	0.499	1.0000	1.683	1.762	0.567
	31	2.13	0.421	0.9989	1.999	2.063	0.484
	31.5	2.54	0.303	0.9992	2.847	2.742	0.364
	32	2.50	0.359	0.9999	2.403	2.497	0.400
	32.5	2.32	0.262	0.9997	3.255	3.382	0.295
	33	2.45	0.248	0.9996	3.476	3.680	0.271
	33.5	2.49	0.122	0.9970	7.074	7.065	0.141
	34	3.16	0.090	0.9972	9.779	10.162	0.098
	34.5	3.41	0.069	0.9999	12.962	13.098	0.076
	35	3.17	0.061	0.9996	14.430	14.742	0.067
5.1-CP-DDT	42.5	2.2	1.098	0.9983	0.771	1.032	0.968
	43	2.39	0.881	0.9972	0.974	1.315	0.760
	43.5	1.93	0.539	0.9981	1.532	2.178	0.459
	44	1.87	0.361	0.9973	2.280	3.450	0.289
	44.5	1.92	0.273	0.9975	3.021	3.885	0.257
	45	2.35	0.205	0.9936	4.157	5.885	0.185
	45.5	2.42	0.156	0.9959	5.506	7.235	0.138
	46	3.08	0.138	0.9956	6.425	8.170	0.122
	46.5	3.15	0.108	0.9976	8.141	9.497	0.105
	47	4.21	0.086	0.9966	10.673	12.168	0.082

References

1. Curole, B. J.; Broussard, W. J.; Nadeem, A.; Grayson, S. M. Dithiol-yne Polymerization: Comb Polymers with Poly (ethylene glycol) Side chains. *ACS Polymers Au.* **2022**, *3* (1), 70-81. DOI: 10.1021/acspolymersau.2c00045.
2. Lai, C. Y.; Hiltner, A.; Baer, E.; Korley, L. T. Deformation of confined poly (ethylene oxide) in multilayer films. *ACS applied materials & interfaces.* **2012**, *4* (4), 2218-2227. DOI: 10.1021/am300240r.
3. Matxinandiarena, E.; Múgica, A.; Zubitur, M.; Zhang, B.; Ladelta, V.; Zapsas, G.; Hadjichristidis, N.; Müller, A. J. The Effect of the Cooling Rate on the Morphology and Crystallization of Triple Crystalline PE-b-PEO-b-PLLA and PE-b-PCL-b-PLLA Triblock Terpolymers. *ACS Applied Polymer Materials.* **2020**, *2* (11), 4952-4963. DOI: 10.1021/acsapm.0c00826
4. Tadokoro, H.; Chatani, Y.; Yoshihara, T.; Tahara, S.; Murahashi, S. Structural studies on polyethers,[-(CH_2) m-O-]$_n$. II. Molecular structure of polyethylene oxide. *Die Makromolekulare Chemie: Macromolecular Chemistry and Physics.* **1964**, *73* (1), 109-127. DOI: 10.1021/ma60035a005.
5. Takahashi, Y.; Tadokoro, H. Structural studies of polyethers,(-(CH_2) mO-) n. X. Crystal structure of poly (ethylene oxide). *Macromolecules.* **1973**, *6* (5), 672-675.
6. Gu, F.; Bu, H.; Zhang, Z. A unique morphology of freeze-dried poly (ethylene oxide) and its transformation. *Polymer.* **2000**, *41* (21), 7605-7609. DOI: 10.1016/S0032-3861(00)00139-7.
7. Godovsky, Y. K.; Slonimsky, G. L.; Garbar, N. M. Effect of molecular weight on the crystallization and morphology of poly (ethylene oxide) fractions. In *Journal of Polymer Science Part C: Polymer Symposia;* Wiley Subscription Services, Inc., A Wiley Company, New York, 1972; pp 1-21.
8. Buckley, C. P.; Kovacs, A. J. Melting behaviour of low molecular weight poly (ethylene-oxide) fractions: I. Extended chain crystals. *In Polymere Aspekte,* Steinkopf, 1975; pp. 44-52.
9. Buckley, C.P.; Kovacs, A.J. Melting behaviour of low molecular weight poly (ethylene-oxide) fractions. *Colloid & Polymer Science.* **1976**, *254*, 695–715. DOI:10.1007/BF01643767.
10. P. H. Geil. *Polymer Single Crystals*, Interscience Publishers, New York, 1963.

11. Marshall, A.; Domszy, R. C.; Teo, H. H.; Mobbs, R. H.; Booth, C. Crystallinity of ethylene oxide oligomers. *European Polymer Journal.* **1981**, *17* (8), 885-893. DOI: 10.1016/0014-3057(81)90194-4.
12. Sun, H.; Yu, D. M.; Shi, S.; Yuan, Q.; Fujinami, S.; Sun, X.; Wang, D.;, Russell, T. P. Configurationally constrained crystallization of brush polymers with poly (ethylene oxide) side chains. *Macromolecules.* **2019**, *52* (2), 592-600. DOI: 10.1021/acs.macromol.8b02265.
13. Kurz, R.; Achilles, A.; Chen, W.; Schäfer, M.; Seidlitz, A.; Golitsyn, Y.; Saalwächter, K. Intracrystalline jump motion in poly (ethylene oxide) lamellae of variable thickness: A comparison of NMR methods. *Macromolecules*. **2017**, *50* (10), 3890-3902. DOI: 10.1021/acs.macromol.7b00843.
14. Schulz, M.; Seidlitz, A.; Kurz, R.; Bärenwald, R.; Petzold, A.; Saalwächter, K.; Thurn-Albrecht, T. The underestimated effect of intracrystalline chain dynamics on the morphology and stability of semi-crystalline polymers. *Macromolecules.* **2018**, *51* (21), 8377-8385. DOI: 10.1021/acs.macromol.8b01102.
15. Schäfer, M.; Wallstein, N.; Schulz, M.; Thurn-Albrecht, T.; Saalwächter, K. Intracrystalline Dynamics in Oligomer-Diluted Poly (Ethylene Oxide). *Macromolecular Chemistry and Physics.* **2020**, *221* (1), 1900393. DOI: 10.1002/macp.201900393.
16. Schulz, M.; Schäfer, M.; Saalwächter, K.; Thurn-Albrecht, T. Competition between crystal growth and intracrystalline chain diffusion determines the lamellar thickness in semi-crystalline polymers. *Nature Communications.* **2022**, *13* (1), 119. DOI: 10.1038/s41467-021-27752-0.
17. Wang, Z.; Schaller, M.; Petzold, A.; Saalwächter, K.; Thurn-Albrecht, T. How entanglements determine the morphology of semi-crystalline polymers. *Proceedings of the National Academy of Sciences.* **2023**, *120* (27), e2217363120. DOI: 10.1073/pnas.2217363120.
18. Flory, P. J. Thermodynamics of crystallization in high polymers. IV. A theory of crystalline states and fusion in polymers, copolymers, and their mixtures with diluents. *Journal of Chemical Physics.* **1949**, *17*, 223–240. DOI: 10.1063/1.1747230.
19. Flory, P. J.; Vrij, A. Melting points of linear-chain homologs. The normal paraffin hydrocarbons. *Journal of American Chemical Society.* **1963**, *85*, 3548–3553. DOI: 10.1021/ja00905a004.

20. Hu, W. The melting point of chain polymers. *J. Chem. Phys.* **2000**, *113*, 3901–3908.
21. Höhne, G. W. Another approach to the Gibbs–Thomson equation and the melting point of polymers and oligomers. *Polymer.* **2002**, *43*, 4689–4698. DOI: 10.1016/S0032-3861(02)00305-1.
22. W. Thomson. On the equilibrium of vapour at a curved surface of liquid, Lond. Edinb. Dublin Philos. *Mag. J. Sci.* **1871,** *42*, 448–452.
23. Wunderlich, B.; Czornyj, G. A study of equilibrium melting of polyethylene. *Macromolecules.* **1977**, *10*, 906–913. DOI: 10.1021/ma60059a006.
24. Wu, T.; Leng, X.; Wang, Y.; Wei, Z.; Li, Y. Linear-and star-brush poly (ethylene glycol)s: Synthesis and architecture-dependent crystallization behavior. *Polymer.* **2020**, *202*, 122661. DOI: 10.1016/j.polymer.2020.122661.
25. Sun, H.; Yu, D. M.; Shi, S.; Yuan, Q.; Fujinami, S.; Sun, X.; Wang, D.;, Russell, T. P. Configurationally constrained crystallization of brush polymers with poly (ethylene oxide) side chains. *Macromolecules.* **2019**, *52* (2), 592-600. DOI: 10.1021/acs.macromol.8b02265.
